# Taking a trip to the shelf: Behavioral decisions are mediated by the proximity to foraging habitats in the black‐legged kittiwake

**DOI:** 10.1002/ece3.3700

**Published:** 2017-12-10

**Authors:** Signe Christensen‐Dalsgaard, Roel May, Svein‐Håkon Lorentsen

**Affiliations:** ^1^ Department of Biology Norwegian University of Science and Technology (NTNU) Trondheim Norway; ^2^ Norwegian Institute for Nature Research (NINA) Trondheim Norway

**Keywords:** central‐place foragers, foraging decisions, GPS tracking, resource allocation, seabird

## Abstract

For marine top predators like seabirds, the oceans represent a multitude of habitats regarding oceanographic conditions and food availability. Worldwide, these marine habitats are being altered by changes in climate and increased anthropogenic impact. This is causing a growing concern on how seabird populations might adapt to these changes. Understanding how seabird populations respond to fluctuating environmental conditions and to what extent behavioral flexibility can buffer variations in food availability can help predict how seabirds may cope with changes in the marine environment. Such knowledge is important to implement proper long‐term conservation measures intended to protect marine predators. We explored behavioral flexibility in choice of foraging habitat of chick‐rearing black‐legged kittiwakes *Rissa tridactyla* during multiple years. By comparing foraging behavior of individuals from two colonies with large differences in oceanographic conditions and distances to predictable feeding areas at the Norwegian shelf break, we investigated how foraging decisions are related to intrinsic and extrinsic factors. We found that proximity to the shelf break determined which factors drove the decision to forage there. At the colony near the shelf break, time of departure from the colony and wind speed were most important in driving the choice of habitat. At the colony farther from the shelf break, the decision to forage there was driven by adult body condition. Birds furthermore adjusted foraging behavior metrics according to time of the day, weather conditions, body condition, and the age of the chicks. The study shows that kittiwakes have high degree of flexibility in their behavioral response to a variable marine environment, which might help them buffer changes in prey distribution around the colonies. The flexibility is, however, dependent on the availability of foraging habitats near the colony.

## 1 INTRODUCTION

Marine habitats worldwide are undergoing rapid changes due to increased anthropogenic impacts, including overfishing, climate change, pollution, and coastal development (Crain, Kroeker, & Halpern, 2008; Game et al., 2009; Halpern et al., 2008). These changes can have important influence on marine biodiversity and food webs from primary producers to top predators (e.g., Poloczanska et al., 2013) and have increased the conservation concern of many populations of marine top predators such as seabirds (e.g., Croxall et al., 2012; Lescroël et al., 2016; Lewison et al., 2012). Understanding behavioral flexibility, how animals adjust their behavior with respect to temporal and spatial variation in their environment at multiple scales, is important to predict how they might be able to adapt to future environmental changes (Grémillet & Boulinier, 2009; Nussey, Wilson, & Brommer, 2007). Flexibility in foraging behavior is pivotal to buffer spatial and temporal variations in food availability and abundance (Pettex et al., 2012). Hence, to implement proper long‐term conservation measures intended to protect marine top predators, it is crucial to understand their foraging flexibility and how this is related to the oceanographic (e.g., Daunt et al., 2002; Pettex et al., 2012; Votier et al., 2010) and environmental conditions (Lescroël et al., 2016; Lewis, Phillips, Burthe, Wanless, & Daunt, 2015; Yamamoto et al., 2016) that surround them.

During the breeding season, seabirds are central‐place foragers with foraging ranges limited by the need to return to the colony at regular intervals to provision their chick(s) (Orians & Pearson, 1979). The provisioning of growing chicks is a particular energy‐demanding stage of the breeding season (Drent & Daan, 1980), when adults have to balance their resource allocation between maintaining their own body condition and the needs of their offspring (Erikstad, Fauchald, Tveraa, & Steen, 1998). In this period, adults face a trade‐off between increased investments in the current reproductive event and their own chances to survive and reproduce in the future (e.g., Stearns, 1992) and, thus, must evaluate multiple factors to optimize their foraging decisions. Marine ecosystems are dynamic with high spatio‐temporal variability in prey availability. The foraging behavior and choice of habitat of seabirds is, thus largely dependent on the distribution, abundance, and predictability of their prey (Staniland, Trathan, & Martin, 2006). At larger spatial scales, prey is often concentrated in association with specific marine features such as seamounts, fronts, shelf breaks, or eddies, and predators can therefore increase their foraging efficiency by choosing to forage in such areas (Fauchald, 2009; Weimerskirch, 2007). Associations between predator and prey can, however, also be driven by other mechanisms, for example, diurnal cycles of light intensity (van der Kooij, Scott, & Mackinson, 2008), tidal rhythm (Drew, Piatt, & Hill, 2013; Irons, 1998), or wind speed and direction (Garthe, Markones, Hüppop, & Adler, 2009; Lewis et al., 2015). Furthermore, foraging behavior can be determined by intrinsic factors such as adult body condition (Weimerskirch, 1998) and breeding stage and age of chicks (Robertson, Bolton, Grecian, & Monaghan, 2014); further, the sex of the birds may result in different foraging behavior (e.g., Lewis et al., 2002; Lorentsen, 1996; Weimerskirch, Le Corre, Ropert‐Coudert, Kato, & Marsac, 2006). Flexibility in foraging strategies in response to fluctuations in prey availability around the colony throughout the breeding season has been documented for many seabirds (e.g., Ponchon et al., 2014; Suryan et al., 2002; Weimerskirch, 1998; Welcker, Beidersdorf, Varpe, & Steen, 2012). There has, however, been little focus on what drives the behavioral choices of individual foraging trips and the role of environmental predictability in shaping these foraging decisions.

**Figure 1:**
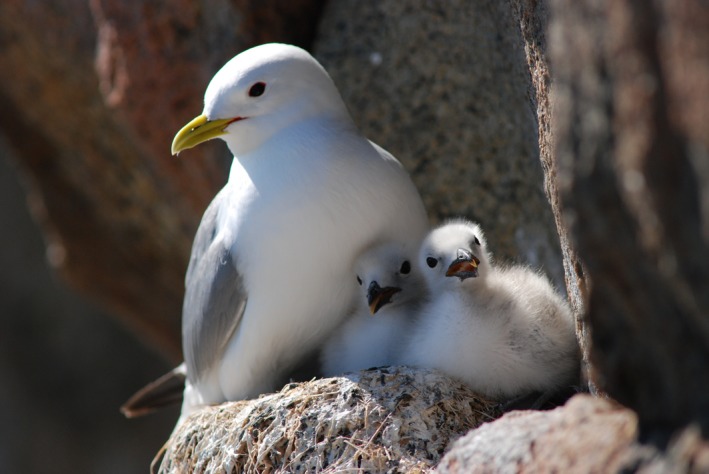
Breeding black‐legged kittiwake (*Rissa tridactyla)* with two chicks at the colony on Anda

**Figure 2:**
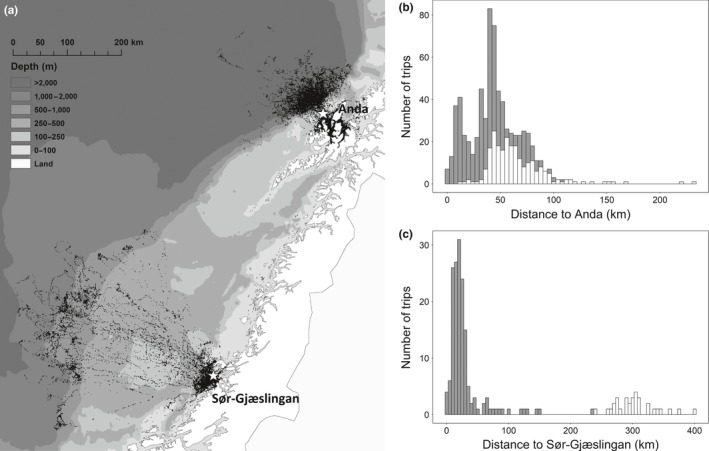
Map of Norway and the study areas, with the colonies marked with white stars (a). Shades of gray show the water depth around the colonies, with light gray being the shallowest. Black dots represent 1 × 1 km squares where foraging behavior was recorded by GPS‐loggers. The graphs show the distribution of maximum foraging distance of individual foraging trips at Anda (b) and Sør‐Gjæslingan (c). Dark gray bars represent fjord trips and white bars oceanic trips. Note the difference in scale on the two graphs

In this study, we explored the behavioral flexibility of breeding black‐legged kittiwakes *Rissa tridactyla* (hereafter kittiwake, Figure 1), a widely distributed small pelagic surface‐feeding seabird, breeding in colonies throughout the Northern Hemisphere. Kittiwakes are sensitive to variations in food availability because they, as surface feeders, rely on crustaceans or fish being available near the surface (Monaghan, 1996). Thus, they have limited capacities to switch to alternative prey (Furness & Tasker, 2000; Piatt et al., 2007), and to buffer environmental variability (Monaghan, 1996). Proximity to predictable foraging areas, where prey is being made available by biological forcing or vertical migration, is therefore expected to be especially important for this species (Byrd, Schmutz, & Renner, 2008; Paredes et al., 2012). The waters off the coast of Norway are highly productive due to favorable oceanographic conditions linked to the two north‐flowing currents, the Norwegian Coastal Current close to shore and the North Atlantic Current, which transport warm saline water along the continental shelf (Barrett, Lorentsen, & Anker‐Nilssen, 2006; Skjoldal, Dalpadado, & Dommasnes, 2004). This creates a productive frontal zone (the coastal front) following the edge of the continental shelf (Rey, 2004), with predictable food prey availability to seabirds. Off the coast of central Norway, the continental shelf is wide, and the frontal areas at the shelf break are situated more than 300 km from the coast, whereas off the coast of northern Norway, near Lofoten and Vesterålen, the shelf is narrow with the edge approaching the coast to within 10 km (Figure 2).

In this study, we examined how the distribution of foraging habitats within the foraging range of breeding kittiwakes, and especially proximity to the predictable and productive frontal zone, shapes their foraging decisions and impacts their foraging behavior. Using data from two Norwegian kittiwake colonies with large differences in the distance to the frontal areas at the shelf break, we reduced the complex mechanisms of foraging habitat choice in a heterogeneous marine environment to a binary choice of foraging either at the shelf break or in nearby coastal waters. We assessed which intrinsic and extrinsic factors affect the choice for each foraging habitat, and the birds’ respective behavior in those habitats, using data from a combination of GPS‐, TDR‐, and GLS‐loggers tracking chick‐rearing kittiwakes during multiple years. Our central research questions were as follows: (1) Which extrinsic and intrinsic factors affect the choice of foraging habitat? (2) How do between‐colony differences in proximity to foraging habitats affect foraging decisions?, and (3) How is kittiwake behavior during foraging trips influenced by extrinsic and intrinsic factors in the respective foraging habitats?

## 2 MATERIAL AND METHODS

### 2.1 Study system

The study was conducted during the chick‐rearing period in 2011–2014 at two Norwegian colonies: Sør‐Gjæslingan (64°44′N, 10°46′E) in the central Norwegian Sea, situated ca. 300 km from the shelf break, and Anda (69°03′N, 15°10′E) in the northern Norwegian Sea, situated ca. 30 km from the shelf break (Figure 2). The kittiwake population on Anda is one of few populations on the Norwegian mainland that have remained stable during the last decade (Anker‐Nilssen et al., 2017), whereas the colony on Sør‐Gjæslingan has been decreasing during recent years (Christensen‐Dalsgaard, unpublished data). During the study period, a mean of 898 and 423 pairs nested on Anda and Sør‐Gjæslingan, respectively.

### 2.2 Field data collection

Birds were captured on the nest using a noose pole or a noose trap. The bird's head was covered during handling to reduce stress. Body mass was measured with a spring balance (Pesola ± 1 g), and GPS‐loggers were deployed on 314 randomly chosen breeding adults rearing chicks 1–24 days of age. We used mGPS‐2 GPS‐loggers from earth&OCEAN Technologies, or i‐gotU GT‐120 GPS‐loggers from MobileAction, disassembled from their outer casing and refitted with a smaller battery to reduce weight. The number of chicks in the nest was recorded, and the chicks were aged based on visual examination and comparison with chicks of known age. In 2013 and 2014, birds on Anda were, in addition to the GPS‐loggers, fitted with a Mk18H (British Antarctic Survey) or MK4083 (Biotrack) geolocator‐immersion logger (hereafter GLS‐logger), or with a LAT1900 (Lotek) temperature and depth data logger (hereafter TDR‐logger).

GPS‐loggers were attached to three or four feathers on the rump of the birds using white *Tesa* tape (Beiersdorf, Germany) and were configured to record a location at 60‐ or 120‐s intervals. The GLS‐ and TDR‐loggers were attached to a Darvic ring with cable tie secured with super glue (Loctite) and fastened on the tarsus of the bird. GLS‐loggers recorded saltwater immersions every 3 s, and data were stored as the total number of wet events during a 10‐min period (completely dry = 0, completely wet = 200). The TDR‐loggers were configured to record pressure and saltwater immersion every second. Total mass of loggers used in the study was 12 g and 13 g when only a GPS‐logger was deployed, 14.9 g when GPS‐ and GLS‐loggers were deployed, and 15.5 g when GPS‐ and TDR‐loggers were deployed. This equaled 3.5% (360 g, *N = *107, *SE* = 2.76), 3.5% (371 g, *N = *96, *SE* = 3.42), 4.0% (371 g *N = *90, *SE* = 3.76), and 4.2% (373 g, *N = *21, *SE* = 6.87) of the mean weight of the birds for each of the respective logger combinations. Capture and handling of an individual took on average 12.4 min (*SE* = 0.20, *n* = 286). Instrumented individuals were recaptured after 2–4 days, and the loggers were removed. At recapture, body mass and skull length (head and bill; using a slide calliper, accuracy to 0.01 mm) were measured, and blood (25 μl) was sampled for sexing.

During the study period, in total 294 GPS‐loggers were recovered (*n* deployed/recovered: Sør‐Gjæslingan 124/115 and Anda 190/179), giving a recovery rate of 94%. Loggers not recovered were caused by the birds losing the loggers before it was possible to recapture them. This resulted in data from 914 complete trips (705 from Anda and 209 from Sør‐Gjæslingan).

### 2.3 Data screening of foraging trips

Following Paredes et al. (2012), a maximum speed of 80 km/h was used to filter out locations, as they were likely to have been caused by locational error. The following variables were calculated to summarize the trip metrics of each individual: (1) trip duration, defined as the time spent from when the individual left the colony to when it returned; (2) maximum foraging distance, defined as the straight‐line distance between the nest site and the most distant location during the foraging trip; (3) total trip length travelled, calculated as the summed distance between all GPS‐locations from each trip. The foraging trip was considered complete if the bird returned to within 1 km of the colony. Long time gaps when satellite reception was lost did, however, occur, affecting the calculation of trip metrics. When complete trips were not recorded, we included a maximum and total distance estimate in our analysis if the individual had departed or returned to within 50% of the distance between the first or last group of GPS‐locations with feeding activity and the colony on the specific trip before the GPS‐locations were lost. The missing part of the path was extrapolated directly back to the colony using the mean flight speed during the existing part of the out‐ or inward part of the trip. In total, 3.6% of the trips were removed due to incompleteness.

Some of the foraging trips of birds from Anda go into winding fjords and straits. GPS‐locations have shown that birds seldom fly over land. Thus, the actual maximum distance from the colony of these trips is systematically longer than the straight‐line distance between the nest site and the most distant location during the foraging trip (as defined above). To correct for this, we used the ArcGIS CostDistance function (ArcGIS 10.1) to calculate the least‐cost path (the shortest path following only 5 × 5 m grids assigned as sea) between each GPS‐location to the colony. The GPS‐location identified as being furthest away from the colony following the least‐cost path was then used as the maximum foraging distance. The use of grids in the CostDistance function can cause a slight overestimation of distance compared to true distance. To account for this, least‐cost paths were created for trips with no obstructions. The distances obtained using this method were then compared to the actual distances, which showed a mean overestimation of distance of 3.90% (*SE* = 0.14). The distances calculated using least‐cost paths were therefore reduced accordingly.

### 2.4 Foraging habitats, behavioral states, and explanatory variables

Foraging trips were assigned to foraging habitats based on where the location furthest away from the colony was situated, with “oceanic” representing frontal systems at the shelf break and “coastal” representing feeding areas along the coast. On Sør‐Gjæslingan, there was a clear bimodal distribution in the length of maximum foraging distance, separating “coastal” trips (<200 km) from oceanic trips (≥200 km, Figure 2). On Anda, the distinction was based on visual inspection on whether the birds travelled into the fjords or to the shelf break.

Identification of behavioral states during the foraging trips was performed using a multivariate clustering method based on *k*‐means clustering. Commonly used and computationally simple, *k*‐means is a straightforward and effective method of assigning bouts of animal behavior to clusters by fitting the observations into groups with similar traits (Van Moorter, Visscher, Jerde, Frair, & Merrill, 2010). To identify the number of clusters in the data set, gap statistics using a *k*‐means clustering were applied, using the variables speed, change in speed, and absolute turning angles (Tibshirani, Walther, & Hastie, 2001; Van Moorter et al., 2010). To measure the predictability and stability of the *k*‐means cluster assignment, three different cross‐validations were conducted based on randomized, cumulative, and spatio‐temporal partitioning (see Appendix S1). Information on saltwater immersion from TDR‐ and GLS‐loggers was used to validate the precision in identification of groups. The three behavioral clusters resting, commuting, and foraging were identified and used as variables in the analysis (see Appendix S1 for further description and validation of method).

**Figure 3:**
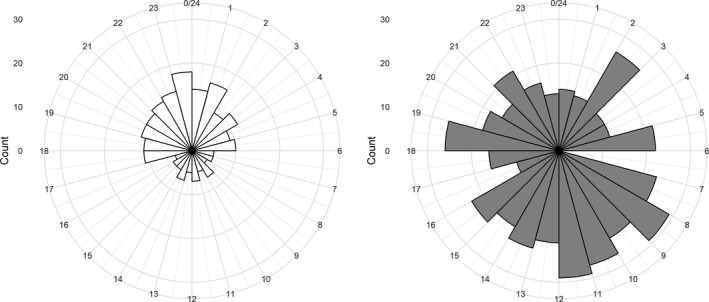
Counts of oceanic (left) and coastal (right) foraging trips of kittiwakes from Anda during the 24‐hr cycle of the day. The length of the bars in each figure depicts the total number of trips conducted in each hourly period

Information on direction and speed of wind at Nordøyan (13 km from Sør‐Gjæslingan) and Andøya (46 km from Anda) at hourly intervals was downloaded from the Norwegian Meteorological Institute (http://www.eklima.no). The wind direction was subsequently divided into three groups, northeasterly (NE, 0°–120°), southerly (S, 120°–240°), and northwesterly (NW, 240°–360°) based on the prevailing wind directions during foraging trips (Figures S2.1 and S2.2). Information on tidal stage (ebb or flood) was downloaded from The Norwegian Mapping Authority (http://www.kartverket.no/sehavniva/). The time when the tracked kittiwake departed the colony on a foraging trip was included, rounded to every whole hour between 0 and 23. Based on a prescreening of the data (see Figure 3), time of departure showed a cyclic pattern with extremes centered on 11:30 (minimum) and 23:30 (maximum). To account for this circularity of the time of departure, hourly values were transformed by (1−Cos((Hour+0.5)·15)/180·π)/2 rendering values ranging between 0 (midnight) and 1 (noon). An index of body condition (BCI) of the instrumented kittiwakes was estimated using the residuals from a regression of body mass on total head and bill length (Kristensen et al., 2012; Schulte‐Hostedde, Zinner, Millar, & Hickling, 2005). BCI was calculated separately for males and females as males tend to be larger than the females (see also Barrett, Fieler, Anker‐Nilssen, & Rikardsen, 1985). Age of the chicks of the instrumented birds was also included as a variable (Table 1).

**Table 1:** Description and grouping of variables used in the analysis of factors potentially affecting the foraging behavior of kittiwakes

	Variable name	Description	Statistics Anda	Statistics SG
Random factor	Bird ID	Individual identification code	171	81
	Year	Year that the trip took place	2011: 70	2011: 17
			2012: 33	2012: 41
			2013: 289	2013: 88
			2014: 313	2014: 63
Time‐related extrinsic variables	Departure time (DT)	Continuous, indicating time of departure in whole hours	min: 0, max: 23	min: 0, max: 23
	Tidal phase	Factor, describing whether it is ebb or flood	ebb: 337	ebb: 103
			flood: 368	flood: 106
	Proportion ebb (PE)	Continuous, proportion of locations per trip with ebb	mean: 0.5	mean: 0.5
Wind‐related extrinsic variables	Wind speed (WS)	Continuous, indicating average wind speed in m/s‐1	mean: 4.2	mean: 7.2
			max: 11.1	max: 14.3
	Wind direction (WD)	Factor: NW, S and NE	NW: 270	NW: 86
			S: 161	S: 38
			NE: 274	NE: 77
Intrinsic variables	Chick age (CA)	Continuous, age in days of the chick(s) of the instrumented birds	min: 1, max: 25	min: 1, max: 22
	Sex	Sex of the bird	male: 348, female: 357	male: 115, female: 94
	BCI	Continuous, index of body condition	mean male: −3.4, mean female: −1.7	mean male: −3.4, mean female: −11.6

The statistics are based on number of trips registered on Anda and Sør‐Gjæslingan (SG) in 2011–2014.

Weather conditions at the time of departure of each trip were used in the analysis of habitat selection, as this was considered the moment when the birds made the decision on where to forage. For the analysis of behavior during the foraging trips, the value of each variable at every GPS‐location constituting the trip was averaged across the whole trip.

### 2.5 Model selection and statistical analysis

Choice of habitat (oceanic versus coastal) and behavior during foraging trips were modeled using generalized linear mixed models (GLMMs) with binomial error distribution (Zuur, Ieno, Walker, Saveliev, & Smith, 2009). In all mixed model analyses, bird ID nested within year was included as random intercept to account for pseudoreplication. Analyses were carried out using the R package lme4 (Bates, Mächler, Bolker, & Walker, 2015). The Akaike's information criterion (AIC) was used for model selection (Burnham, Anderson, & Huyvaert, 2011).

We constructed 13 candidate models to assess habitat choice and trip behavior, utilizing the fixed effects presented in Table 1. We included interacting effects when biologically feasible, but restricted this to two‐way interactions to reduce the number of parameters to be estimated. As we expected wind speed to influence the availability of prey to kittiwakes, we considered possible interacting effects of wind speed with time of departure from the colony, BCI, and age of chicks, in addition to an interacting effect of wind speed and direction. We also expected a possible interacting effect of BCI and sex. To assess the relative importance of intrinsic and extrinsic factors in determining habitat choice and trip behavior, grouped AIC weights were calculated for models including the following variables: intrinsic (age of chicks, BCI, and sex), extrinsic weather variables (wind speed and direction), and extrinsic time‐related variables (departure time, tidal phase, and proportion of ebb) (Table 1).

## 3 Results

### 3.1 Selection of foraging habitat

Despite the path length being, on average, four times as long for kittiwakes foraging in the oceanic habitat at Sør‐Gjæslingan compared to Anda (Table 2), individual birds from both colonies used both habitat types. All years combined, most foraging trips were in coastal foraging habitat (Table 2, Table S4.1). Nevertheless, the model selection procedure revealed different factors affecting choice of foraging habitats at the two colonies (Table 3).

**Table 2:** Statistics (mean ± *SE*) of the different trip parameters and distribution of behaviors during foraging trips on Anda and Sør‐Gjæslingan for all years combined

	Locality	Variable	Coastal	Oceanic
Trip characteristics	Anda	Duration (h)	6.4 ± 0.23	7.4 ± 0.26
		Path length (km)	100.0 ± 2.90	201.0 ± 5.62
		Maximum distance (km)	36.7 ± 0.97	63.5 ± 1.68
		Number of trips (%)	68	32
	Sør‐Gjæslingan	Duration (h)	5.66 ± 0.30	29.0 ± 2.29
		Path length (km)	100.2 ± 6.38	795.6 ± 42.66
		Maximum distance (km)	27.66 ± 2.38	303.7 ± 6.06
		Number of trips (%)	84	16
Behavior	Anda	Resting (%)	45.0 ± 0.70	11.7 ± 0.70
		Foraging (%)	29.1 ± 0.50	31.3 ± 0.63
		Commuting (%)	25.8 ± 0.55	57.0 ± 0.84
	Sør‐Gjæslingan	Resting (%)	37. 8 ± 1.58	21.4 ± 2.20
		Foraging (%)	32.1 ± 0.97	20.9 ± 0.95
		Commuting (%)	30.1 ± 1.20	57.7 ± 2.43

**Table 3:** Model selection for choice of habitat by birds from Anda and Sør‐Gjæslingan, ordered by the AIC from Anda

Model	*df*	Anda			Sør‐Gjæslingan		
		AIC	ΔAIC	*w*_*i*_	AIC	ΔAIC	*w*_*i*_
Departure time*wind speed	6	**768.3**	**0**	**1.00**	173.5	11.8	0.00
Day number*departure time	6	780.3	12	0.00	173.0	11.3	0.00
Departure time	4	781.9	13.6	0.00	169.9	8.2	0.01
BCI* wind speed	6	822.0	53.7	0.00	162.0	2.3	0.22
Day number*wind speed	6	824.1	55.8	0.00	173.3	11.6	0.00
Wind speed	4	824.2	55.9	0.00	169.6	7.9	0.01
Wind direction*wind speed	8	829.9	61.6	0.00	172.7	11.0	0.00
Day number	4	847.4	79.1	0.00	169.9	8.2	0.01
BCI	4	845.0	81.7	0.00	**161.7**	**0**	**0.42**
Tide	4	851.4	83.1	0.00	167.9	6.2	0.02
0‐model	3	852.8	84.5	0.00	167.9	6.2	0.02
BCI*sex	6	853.8	85.5	0.00	164.0	2.3	0.13
Sex	4	854.6	86.3	0.00	163.9	2.2	0.14
Wind direction	5	856.0	87.7	0.00	168.5	6.8	0.01

The models with the lowest AIC shown for each site are shown in bold. *df*, degrees of freedom; AICc, AIC corrected for finite sample size; ΔAICc, difference between the AICc of the model and the best model; *w*_*i*_, ratio of AICc values for this model relative to the whole set of candidate models (weight).

**Figure 4:**
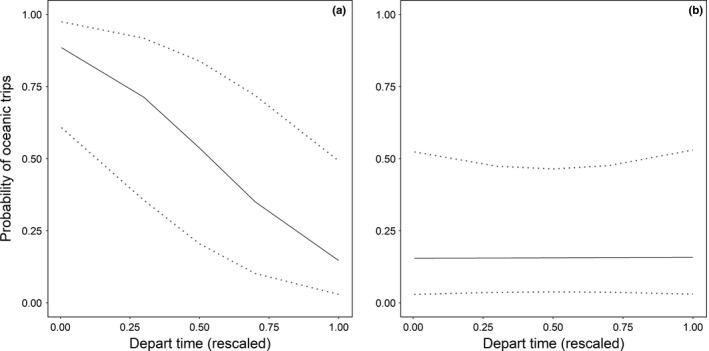
Predicted probabilities from the best model describing habitat selection of kittiwakes on Anda. Probability of conducting oceanic trips when departing at different times of the day is shown for (a) no wind (0 ms^1^) and (b) strong winds (10 ms^1^). The values on the *x*‐axis are rescaled values, ranging between 0 (midnight) and 1 (noon)

The most supported model explaining choice of foraging habitat (oceanic versus coastal) for kittiwakes on Anda included time of departure from the colony, wind speed, and their interaction term (Table 3). There was diurnal pattern in habitat choice, with significantly less birds foraging in the oceanic habitats during daytime compared to the coastal habitat (β = −3.8 ± 0.74, *p *<* *.001, Figure 3). Wind speed had a negative effect on the probability of conducting oceanic trips, with more birds foraging in the coastal habitat during strong winds (β = −4.2 ± 1.02, *p *<* *.001, Figure 4). Furthermore, the probability of foraging in the coastal habitats during the night increased with increasing wind speeds (β_interaction_ = 4.3 ± 1.69, *p *=* *.012, Figure 4).

**Figure 5:**
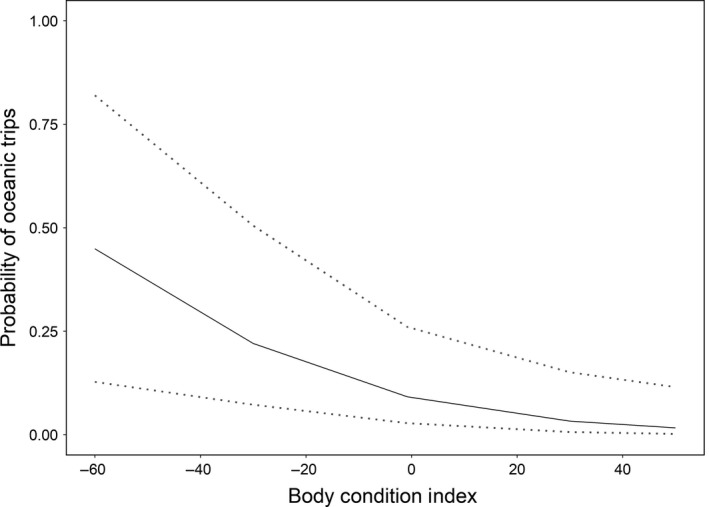
Predicted probabilities from the best model describing habitat selection of kittiwakes on Sør‐Gjæslingan. The probability of conducting oceanic trips is shown as function of body condition index

On Sør‐Gjæslingan, the highest ranked model included the BCI of birds (Table 3). The model showed that choice of oceanic foraging habitat was related to the BCI of the individual, with birds having a low BCI showing higher probability of using oceanic foraging habitats (best model: β = −3.9 ± 1.47, *p *=* *.008, Figure 5).

### 3.2 Factors affecting behavior during foraging trips

Independent of study colony, birds used similar proportions of time commuting in respectively oceanic and coastal foraging habitats (Table 2). On Anda, birds used similar proportions of time foraging in the coastal and the oceanic habitats, but about four times as much time resting in the coastal habitat compared to the oceanic. On Sør‐Gjæslingan, birds spent similar proportions of time foraging and resting in coastal and oceanic foraging habitats (Table 2).

For kittiwakes on Anda, the proportion of commuting in both foraging habitats was best explained by departure time from the colony, age of chicks, and their interaction term (Table 4 and Appendix S3.1). When having young chicks, birds commuted equally throughout the day, but as the chicks aged, birds commuted less during the middle of the day. The proportion of resting in both habitats was best explained by the same model as for commuting, albeit with opposite direction. The models explaining foraging behavior differed between habitats (Table 4 and Appendix S3.1). For foraging in the oceanic habitat, two models were supported. The highest ranked model included departure time, wind speed, and their interaction term, whereas the second‐best model included departure time, age of the chick, and their interaction term (ΔAIC = 1.44). In oceanic habitat, birds displayed more foraging behavior with increasing wind speeds but less when departing later during the day unless at higher wind speeds. For foraging behavior in the coastal habitat, the best model included wind speed, age of chicks, and their interaction term. Thus, in the coastal habitat, the proportion of time spent engaged in foraging behavior increased with wind speed but decreased with the age of chicks.

**Table 4:** Effect sizes and significance level for most parsimonious models explaining behavior during oceanic and coastal trips at the Anda and Sør‐Gjæslingan (SG) colonies

Behavior	Colony	Habitat	*n*	Effect size (*z*‐value)											
				DT	WS	WD^a^		CA	BCI	DT:CA	DT:WS	WS:CA	WS:WD^a^		BCI:WS
						NW	S						NW	S	
Commuting	Anda	Coastal	18969	0.55				0.10		−3.06**					
Commuting	Anda	Oceanic	24379	2.50*				2.96**		−6.28 ***					
Foraging	Anda	Coastal	21521		4.16***			−2.64**				−0.70			
Foraging	Anda	Oceanic	13581	−3.43***	0.65						1.76				
Resting	Anda	Coastal	38691	−2.65**				0.88		5.49***					
Resting	Anda	Oceanic	5909	2.57*				−0.59		5.03***					
Commuting	SG	Coastal	7278		2.95**	−0.02	−0.57						2.69**	1.92	
Commuting	SG	Oceanic	12471		1.21				2.64**						−2.13*
Foraging	SG	Coastal	7428	0.46				1.97		−5.49***					
Foraging	SG	Oceanic	4531		1.96^b^										
Resting	SG	Coastal	8531	0.50	−0.62						2.32*				
Resting	SG	Oceanic	4474		−2.95**				−1.52						2.39*

Abbreviations are given in Table 1. Stars indicate the level of significance, with *p <* *.05, **p <* *.01 and ***p <* *.001. For full set of candidate models, see Appendix S4.

a Relative to the reference category NE.

b 0‐model < 2Δ.

For kittiwakes on Sør‐Gjæslingan, the proportion of commuting behavior was best explained by wind speed, for oceanic habitat in interaction with BCI, and for coastal habitat in interaction with wind direction (Table 4 and Appendix S3.2). In the oceanic habitat, birds commuted more with increasing wind speed or BCI. However, birds with a higher BCI were less likely to commute at higher winds. In coastal habitat, birds spent more time commuting with increasing winds, but this effect depended on the direction of the wind (less for northeasterly winds). The proportion of time used resting was best explained by wind speed, for oceanic habitat in interaction with BCI and for coastal habitat in interaction with time of departure. In oceanic habitat, resting was directly opposite the result for commuting. In coastal habitat, birds rested more when departing later during the day and with increasing wind speeds. The models explaining foraging behavior differed between habitats (Table 4 and Appendix S3.2). The proportion of foraging behavior in oceanic habitats was best explained by increasing wind speeds. However, the 0‐model differed only 0.776 ΔAIC from the best model, which was therefore disregarded. Foraging behavior in coastal habitat was influenced most by the time when birds departed from the colony, the age of the chicks, and their interaction term. When having young chicks, birds foraged equally throughout the day, but as chicks aged birds foraged less during the middle of the day.

### 3.3 Intrinsic and extrinsic drivers for foraging decisions

While choice of habitat was determined by intrinsic factors on Sør‐Gjæslingan (AIC_w_ = 0.929), it was determined by extrinsic factors on Anda (both weather and time related AIC_w_ = 1, Table 5). Intrinsic factors and weather‐ and time‐related factors affected behavior during foraging trips differently at the two colonies. Still, at both colonies commuting and resting showed the same pattern. Overall, behavior on Anda was best explained by intrinsic (mean AIC_w_ oceanic: 0.766; coastal: 0.873) and time‐related factors (AIC_w_ oceanic: 0.972; coastal: 0.569), and less by weather (AIC_w_ oceanic: 0.233, coastal: 0.441). On Sør‐Gjæslingan, weather (AIC_w_ oceanic: 0.802, coastal: 0.537) and intrinsic factors (AIC_w_ oceanic: 0.668, coastal: 0.394) contributed more to explaining behavior than did time‐related factors (AIC_w_ oceanic: 0.055, coastal: 0.631).

**Table 5:** Summed AIC weights of all candidate models (see Table 3 and Appendix S4) grouped into intrinsic and extrinsic weather‐ and time‐related factors explaining choice of foraging habitat, behavior during foraging trips, as well as averaged over all three behaviors at both colonies

	Anda		Sør‐Gjæslingan	
	Oceanic	Coastal	Oceanic	Coastal
Habitat				
Intrinsic	0.000	0.929		
Weather	1.000	0.153		
Time	1.000	0.030		
Commuting				
Intrinsic	0.998	0.669	0.834	0.010
Weather	0.002	0.323	0.991	0.999
Time	1.000	0.700	0.010	0.000
Foraging				
Intrinsic	0.300	0.950	0.237	1.000
Weather	0.697	0.999	0.421	0.000
Time	0.916	0.008	0.151	1.000
Resting				
Intrinsic	1.000	1.000	0.933	0.171
Weather	0.000	0.000	0.995	0.613
Time	1.000	1.000	0.003	0.894
Behavior				
Intrinsic	0.766	0.873	0.668	0.394
Weather	0.233	0.441	0.802	0.537
Time	0.972	0.569	0.055	0.631

## 4 DISCUSSION

There was a fivefold difference in distance to the feeding area at the shelf break between the two study colonies. The average path lengths were twice and eight times as long, for Anda and Sør‐Gjæslingan, respectively, when birds went to forage at the shelf break compared to coastal habitats. Kittiwakes from both colonies, nonetheless, conducted foraging trips to the shelf break clearly demonstrating its importance as foraging habitat. Foraging theory predicts that animals should travel the minimum distance to meet energy requirements (Schoener, 1971), and when traveling to more distant feeding grounds, the extra cost must therefore be compensated by increased energy gain (Stephens & Krebs, 1986). The frequent use of the foraging habitats at the shelf break might suggest that the difference in prey density and/or availability at the distant foraging habitat outweigh the additional costs of commuting.

The proximity of the colonies to predictable foraging habitats strongly mediated which factors drove decisions to forage at the shelf break contra nearby coastal areas. When breeding close to the shelf break (as exemplified at Anda), the choice of foraging habitat was driven by extrinsic factors whereas body condition of the instrumented birds determined when to conduct long trips out to the shelf break for birds breeding further away from this habitat (as exemplified at Sør‐Gjæslingan). Animals encounter a hierarchy of decisions when optimizing their foraging efforts (Stephens, 2008). Within a heterogeneous environment, seabirds therefore have to be able to adapt their foraging behavior (Hernández‐Pliego, Rodríguez, & Bustamante, 2014) and balance the allocation of resources for reproduction and self‐maintenance (Erikstad et al., 1998).

The kittiwakes at Anda (situated nearest to the shelf break) showed a fine‐tuned flexibility in choice of foraging habitats, primarily dictated by the diurnal patterns of prey availability in the different habitats (Kristoffersen, 1999; van der Kooij et al., 2008), optimizing the net energy gain throughout the diurnal cycle. Kittiwakes from Anda primarily feed on sandeel (*Ammodytes* spp) while in the coastal areas, and mesopelagic fish while in the frontal systems at the shelf break (Christensen‐Dalsgaard, unpublished data). These fish species have opposite responses to the time of the day; sandeels display a diurnal pattern where they emerge from the seabed during the day (van der Kooij et al., 2008), whereas mesopelagic fish aggregate close to the surface during the night (Kristoffersen, 1999). Thus, the kittiwakes seemingly timed their foraging schedule to the temporal and spatial pattern in prey availability, suggesting a memory‐based foraging strategy (Irons, 1998; Montevecchi, Benvenuti, Garthe, Davoren, & Fifeld, 2009; Pettex et al., 2012).

The patterns of behavior during foraging trips could also to some extent be assigned to the behavior of key prey species. Birds from Anda, taking foraging trips to the shelf break, rested more and foraged less in the middle of day, which corresponds with mesopelagic fish being less available at the surface during daytime.

At Sør‐Gjæslingan, birds with low body condition indices apparently compensated for low food availability around the colony by foraging in the more remote habitats at the shelf break with more predictable access to prey. Thus, birds interspaced the long trips to the shelf break with short trips closer to the colony, suggesting a bimodal foraging strategy, allowing parents to intermix long trips to replenish their own body reserves with short trips to frequently feed their chicks (Weimerskirch, 1998; Ydenberg & Davies, 2010). The bimodal foraging strategy has been documented for a number of seabird species (Saraux, Robinson‐Laverick, Le Maho, Ropert‐Coudert, & Chiaradia, 2011; Weimerskirch et al., 1994; Welcker et al., 2012), but so far not conclusively for kittiwakes (but see Paredes et al., 2012; Ponchon et al., 2014). The movement patterns observed at Sør‐Gjæslingan could indicate that food supplies around the colony were not sufficiently available to cover both the costs of reproduction and self‐maintenance. This is supported by the fact that birds with lower BCI had a higher preference for the oceanic habitat. We hypothesize that, as conditions around the colony deteriorated, breeding birds of low BCI could not compensate for low food availability and therefore conducted longer trips to ensure their own body reserves. This supposition corresponded with an observed almost total breeding failure at Sør‐Gjæslingan in the study years, attributed to chicks starving to death and high predation on chicks sitting unsupervised on the nests (Christensen‐Dalsgaard, unpublished data). The observed negative effect of wind speed on probability of feeding at the shelf edge could be related to the increased energetic costs of localizing and capturing prey in strong winds with high waves (Lewis et al., 2015).

Handling and instrumenting seabirds with loggers can raise their foraging costs (Vandenabeele, Shepard, Grogan, & Wilson, 2012), leading to an alteration of their foraging behavior (Heggøy, Christensen‐Dalsgaard, Ranke, Chastel, & Bech, 2015; Vandenabeele, Wilson, & Grogan, 2011). The movement data obtained by tracking studies must, thus, be regarded as approximation to the undisturbed system, and the results should be treated accordingly. In our study we used loggers of comparable size with similar studies (e.g., Chivers et al., 2012; Wakefield et al., 2017), but nonetheless exceeded 3% of the birds’ body mass (c.f. Kenward, 2001; Vandenabeele et al., 2012). Previous work has shown that negative effects of loggers may be more evident in birds with low body condition (Heggøy et al., 2015). As kittiwakes on Sør‐Gjæslingan indeed had a low BCI, we only conducted single‐logger instrumentation here to reduce potentially negative effects of instrumentation, thereby keeping logger mass at an average of 3.5% of the birds’ body mass. However, it cannot be disregarded that our findings could be affected by the increased load associated with instrumentation.

### 4.1 Ecological and managemental implications

In our study, kittiwakes showed a high degree of flexibility in their behavioral response to a variable marine environment. When breeding in proximity to multiple predictable foraging areas, the birds appeared to maximize energy gain by selecting the foraging habitats best suited under the given intrinsic and extrinsic conditions. Our results confirm previous findings that proximity to productive habitats influences the foraging strategy of the birds (Navarro & González‐Solís, 2009; Paiva et al., 2010; Paredes et al., 2012), with strong impacts on breeding success and population trends (Paredes et al., 2012). The study colonies have shown contrasting population trajectories during recent years with Anda having a higher breeding success and a higher population growth than Sør‐Gjæslingan (Anker‐Nilssen et al., 2017 and Christensen‐Dalsgaard, unpublished data). Thus, the results from this study can help explain some of the mechanisms behind these differences in population trajectories. The foraging areas at the shelf break were important for birds at both colonies. However, at Sør‐Gjæslingan chick‐rearing kittiwakes were able to increase foraging effort by traveling to the distant shelf break, only at the risk of chick survival. It therefore appears that, as the distance to the productive area increases, there will be a turning point where the distance is too long to make the trip profitable enough to raise chicks successfully (as also shown in Ponchon et al., 2014). Sandvik et al. (2016) indeed showed that seabird colonies are situated in locations that minimize travel distance between breeding and foraging locations.

We showed that kittiwakes displayed a high degree of intercolony flexibility in foraging behavior. The accessibility of several different types of foraging habitats evidently made the kittiwakes more resilient to changes in prey availability and other extrinsic and intrinsic factors. In addition, foraging areas as far as 300 km from the colony were important for the birds during the breeding season, which are well beyond that reported for this species from most localities (Daunt et al., 2002; Thaxter et al., 2012; but see Paredes et al., 2014; Ponchon et al., 2014). It is, thus, important to consider the availability and usage of habitats within the flight range needed to raise chicks successfully, when implementing conservation measures of marine habitats.

Predictions of weather patterns for the next century show an increase in mean and maximum wind speed in northern Europe (McInnes, Erwin, & Bathols, 2011). The implications of this on seabird survival and reproduction are, however, inconclusive. Elliott et al. (2014) showed that kittiwakes could adjust their foraging behavior to compensate for poor weather, such that chick growth was not affected. This corresponds with our results that the behavior of kittiwakes was indeed affected by wind conditions. When provided with the possibility to forage in the more sheltered fjords, kittiwakes selected for this, presumably to minimize negative effects of strong winds. We did not measure the direct effects of wind conditions on energy expenditure or chick growth and can therefore not elucidate if or how access to multiple predictable habitats can buffer potential detrimental effects of increased wind speed. Given the importance of proximate foraging habitats, anthropogenic activities may further hamper access to these or effectuate barriers forcing kittiwakes to increase flight costs (Masden, Haydon, Fox, & Furness, 2010). This may especially impact seabird colonies situated farther from the shelf break. Gaining a better understanding on the complex interactions between species, their prey and the dynamic environment they inhabit will be crucial to understand to which extent they will be able to cope with the projected changes to marine habitats due to increased anthropogenic impacts.

## CONFLICT OF INTEREST

None declared.

## AUTHORS’ CONTRIBUTIONS

SCD and SHL conceived the ideas and designed methodology; SCD collected the data; SCD and RM analyzed the data; SCD led the writing of the manuscript. All authors contributed critically to the drafts and gave final approval for publication.

## DATA ACCESSIBILITY

Data available from the Dryad Digital Repository: https://doi.org/10.5061/dryad.57nr2

## References

[ece33700-bib-0001] Anker‐Nilssen, T. , Strøm, H. , Barrett, B. , Bustnes, J. O. , Christensen‐Dalsgaard, S. , Descamps, S. , … Systad, G. H. (2017). Key‐site monitoring in Norway 2016, including Svalbard and Jan Mayen. SEAPOP Short Report, 1‐2017. 14 pp. Retrieved from www.seapop.no.

[ece33700-bib-0002] Barrett, R. T. , Fieler, R. , Anker‐Nilssen, T. , & Rikardsen, F. (1985). Measurements and weight changes of Norwegian adult puffins *Fratercula arctica* and Kittiwakes *Rissa tridactyla* during the breeding season. Ringing & Migration, 6, 102–112. https://doi.org/10.1080/03078698.1985.9673865

[ece33700-bib-0003] Barrett, R. T. , Lorentsen, S.‐H. , & Anker‐Nilssen, T. (2006). The status of breeding seabirds in mainland Norway. Atlantic Seabirds, 8(3), 97–126.

[ece33700-bib-0004] Bates, D. , Mächler, M. , Bolker, B. M. , & Walker, S. C. (2015). Fitting Linear mixed‐effects models using lme4. Journal of Statictical Software, 67, 1 https://doi.org/10.18637/jss.v067.i01

[ece33700-bib-0005] Burnham, K. P. , Anderson, D. R. , & Huyvaert, K. P. (2011). AIC model selection and multimodel inference in behavioural ecology: Some background, observations, and comparisons. Behavioral Ecology and Sociobiology, 65, 23 https://doi.org/23-35.10.1007/s00265-010-1029-6

[ece33700-bib-0006] Byrd, G. V. , Schmutz, J. A. , & Renner, H. M. (2008). Contrasting population trends of piscivorous seabirds in the Pribilof Islands: A 30 year perspective. Deep‐Sea Research II, 55, 1846–1855. https://doi.org/10.1016/j.dsr2.2008.04.004

[ece33700-bib-0007] Chivers, L. S. , Lundy, M. G. , Colhoun, K. , Newton, S. F. , Houghton, J. D. R. , & Reid, N. (2012). Foraging trip time‐activity budgets and reproductive success in the black‐legged kittiwake. Marine Ecology Progress Series, 456, 269–277. https://doi.org/10.3354/meps09691

[ece33700-bib-0008] Crain, C. M. , Kroeker, K. , & Halpern, B. S. (2008). Interactive and cumulative effects of multiple human stressors in marine systems. Ecology Letters, 11, 1304–1315. https://doi.org/10.1111/j.1461-0248.2008.01253.x 19046359 10.1111/j.1461-0248.2008.01253.x

[ece33700-bib-0009] Croxall, J. P. , Butchart, S. H. M. , Lascelles, B. , Stattersfield, A. J. , Sullivan, B. , Symes, A. , & Taylor, P. (2012). Seabird conservation status, threats and priority actions: A global assessment. Bird Conservation International, 22, 1–34. https://doi.org/10.1017/S0959270912000020

[ece33700-bib-0010] Daunt, F. , Benvenuti, S. , Harris, M. P. , Dall'Antonia, L. , Elston, D. A. , & Wanless, S. (2002). Foraging strategies of the black‐legged kittiwake Rissa tridactyla at a North Sea colony: Evidence for a maximum foraging range. Marine Ecology Progress Series, 245, 239–247. https://doi.org/10.3354/meps245239

[ece33700-bib-0011] Drent, R. H. , & Daan, S. (1980). The prudent parent: Energetic adjustments in avian breeding. Ardea, 68, 225–252. https://doi.org/10.5253/arde.v68.p225

[ece33700-bib-0012] Drew, G. S. , Piatt, J. F. , & Hill, D. F. (2013). Effects of currents and tides on fine‐scale use of marine habitats in a Southeast Alaska hotspot. Marine Ecology Progress Series, 487, 275–286. https://doi.org/10.3354/meps10304

[ece33700-bib-0013] Elliott, K. H. , Chivers, L. S. , Bessey, L. , Gaston, A. J. , Hatch, S. A. , Kato, A. , … Hare, J. F. (2014). Windscapes shape seabird instantaneous energy costs but adult behavior buffers impact on offspring. Movement Ecology, 2, 17 https://doi.org/10.1186/s40462-014-0017-2 26019870 10.1186/s40462-014-0017-2 PMC4445632

[ece33700-bib-0014] Erikstad, K. E. , Fauchald, P. , Tveraa, T. , & Steen, H. (1998). On the cost of reproduction in long‐lived birds: The influence of environmental variability. Ecology, 79(5), 1781–1788. https://doi.org/10.1890/0012-9658(1998)079[1781:OTCORI]2.0.CO;2

[ece33700-bib-0015] Fauchald, P. (2009). Spatial interaction between seabirds and prey: Review and synthesis. Marine Ecology Progress Series, 391, 139–151. https://doi.org/10.3354/meps07818

[ece33700-bib-0016] Furness, R. W. , & Tasker, M. L. (2000). Seabird‐fishery interactions: Quantifying the sensitivity of seabirds to reductions in sandeel abundance, and identification of key areas for sensitive seabirds in the North Sea. Marine Ecology Progress Series, 202, 253–264. https://doi.org/10.3354/meps202253

[ece33700-bib-0017] Game, E. T. , Grantham, H. S. , Hobday, A. J. , Pressey, R. L. , Lombard, A. T. , Beckley, L. E. , … Richardson, A. J. (2009). Pelagic protected areas: The missing dimension in ocean conservation. Trends in Ecology and Evolution, 24, 360–369. https://doi.org/10.1016/j.tree.2009.01.011 19324450 10.1016/j.tree.2009.01.011

[ece33700-bib-0018] Garthe, S. , Markones, N. , Hüppop, O. , & Adler, S. (2009). Effects of hydrographic and meteorological factors on seasonal seabird abundance in the southern North Sea. Marine Ecology Progress Series, 391, 243–255. https://doi.org/10.3354/meps08170

[ece33700-bib-0019] Grémillet, D. , & Boulinier, T. (2009). Spatial ecology and conservation of seabirds facing global climate change: A review. Marine Ecology Progress Series, 391, 121–137. https://doi.org/10.3354/meps08212

[ece33700-bib-0020] Halpern, B. S. , Walbridge, S. , Selkoe, K. A. , Kappel, C. V. , Micheli, F. , D'Agrosa, C. , … Watson, R. (2008). A global map of human impact on marine ecosystems. Science, 319, 948–952. https://doi.org/10.1126/science.1149345 18276889 10.1126/science.1149345

[ece33700-bib-0021] Heggøy, O. , Christensen‐Dalsgaard, S. , Ranke, P. S. , Chastel, O. , & Bech, C. (2015). GPS‐loggers influence behaviour and physiology in the black‐legged kittiwake *Rissa tridactyla* . Marine Ecology Progress Series, 521, 237–248. https://doi.org/10.3354/meps11140

[ece33700-bib-0022] Hernández‐Pliego, J. , Rodríguez, C. , & Bustamante, J. (2014). Gone with the wind: Seasonal trends in foraging movement directions for a central‐place forager. Current Zoology, 60(5), 604–615. https://doi.org/10.1093/czoolo/60.5.604

[ece33700-bib-0023] Irons, D. B. (1998). Foraging area fidelity of individual seabirds in relation to tidal cycles and flock feeding. Ecology, 79, 647–655. https://doi.org/10.1890/0012-9658(1998)079[0647:FAFOIS]2.0.CO;2

[ece33700-bib-0024] Kenward, R. E. (2001). A manual for wildlife radio tagging. London, UK: Academic Press.

[ece33700-bib-0025] Kristensen, D. L. , Erikstad, K. E. , Reiertsen, T. K. , Moum, T. , Barrett, R. T. , & Jenni‐Eiermann, S. (2012). Are female offspring from a single‐egg seabird more costly to raise? Behavioral Ecology, 24, 136–143. https://doi.org/10.1093/beheco/ars144

[ece33700-bib-0026] Kristoffersen, J. B. (1999). Mesopelagic fish in Norwegian waters: Distribution, life history and genetics. Dr. Scient. thesis. Department of Fisheries and Marine Biology, University of Bergen.

[ece33700-bib-0027] Lescroël, A. , Mathevet, R. , Péron, C. , Authier, M. , Provost, P. , Takahashi, A. , & Grémillet, D. (2016). Seeing the ocean through the eyes of seabirds: A new path for marine conservation? Marine Policy, 68, 212–220. https://doi.org/10.1016/j.marpol.2016.02.015

[ece33700-bib-0028] Lewis, S. , Benvenuti, S. , Dall'Antonia, L. , Griffiths, R. , Money, L. , Sherratt, T. N. , … Hamer, K. C. (2002). Sex‐specific foraging behaviour in a monomorphic seabird. Proceedings of the Royal Society of London, Series B: Biological Sciences, 269, 1687–1693. https://doi.org/10.1098/rspb.2002.2083 12204129 10.1098/rspb.2002.2083 PMC1691079

[ece33700-bib-0029] Lewis, S. , Phillips, R. A. , Burthe, S. J. , Wanless, S. , & Daunt, F. (2015). Contrasting responses of male and female foraging effort to year‐round wind conditions. Journal of Animal Ecology, 84, 1490–1496. https://doi.org/10.1111/1365-2656.12419 26283625 10.1111/1365-2656.12419 PMC4989534

[ece33700-bib-0030] Lewison, R. , Oro, D. , Godley, B. , Underhill, L. , Bearhop, S. , Wilson, R. , … Yorio, P. (2012). Research priorities for seabirds: Improving seabird conservation and management in the 21st century. Endangered Species Research, 17, 93–121. https://doi.org/10.3354/esr00419

[ece33700-bib-0031] Lorentsen, S. H. (1996). Regulation of food provisioning in the Antarctic petrel *Thalassoica antarctica* . Journal of Animal Ecology, 65, 381–388. https://doi.org/10.2307/5884

[ece33700-bib-0032] Masden, E. A. , Haydon, D. T. , Fox, A. D. , & Furness, R. W. (2010). Barriers to movement: Modelling energetic costs of avoiding marine wind farms amongst breeding seabird. Marine Pollution Bulletin, 60, 1085–1092. https://doi.org/10.1016/j.marpolbul.2010.01.016 20188382 10.1016/j.marpolbul.2010.01.016

[ece33700-bib-0033] McInnes, K. L. , Erwin, T. A. , & Bathols, J. M. (2011). Global Climate Model projected changes in 10 m wind speed and direction due to anthropogenic climate change. Atmospheric Science Letters, 12, 325–333. https://doi.org/10.1002/asl.341

[ece33700-bib-0034] Monaghan, P. (1996). Relevance of the behaviour of seabirds to the conservation of marine environments. Oikos, 77, 227–237. https://doi.org/10.2307/3546061

[ece33700-bib-0035] Montevecchi, W. A. , Benvenuti, S. , Garthe, S. , Davoren, G. , & Fifeld, D. A. (2009). Flexible foraging tactics by a large opportunistic seabird preying on forage‐ and large pelagic fishes. Marine Ecology Progress Series, 385, 295–306. https://doi.org/10.3354/meps08006

[ece33700-bib-0036] Navarro, J. , & González‐Solís, J. (2009). Environmental determinants of foraging strategies in Cory's shearwater *Calonectris diomedea* breeding on the Canary Islands, NE Atlantic. Marine Ecology Progress Series, 378, 259–267. https://doi.org/10.3354/meps07880

[ece33700-bib-0037] Nussey, D. H. , Wilson, A. J. , & Brommer, J. E. (2007). The evolutionary ecology of individual phenotypic plasticity in wild populations. Journal of Evolutionary Biology, 213, 2365–2371. https://doi.org/10.1111/j.1420-9101.2007.01300.x 10.1111/j.1420-9101.2007.01300.x 17465894

[ece33700-bib-0038] Orians, G. H. , & Pearson, N. E. (1979). On the theory of central place foraging In Horn J., Stairs G. R., & Mitchell R. D. (Eds.), Analysis of ecological systems (pp. 155–177). Columbus, OH: Ohio State University Press.

[ece33700-bib-0039] Paiva, V. H. , Geraldes, P. , Ramírez, I. , Meirinho, A. , Garthe, S. , & Ramos, J. A. (2010). Foraging plasticity in a pelagic seabird species along a marine productivity gradient. Marine Ecology Progress Series, 398, 259–274. https://doi.org/10.3354/meps08319

[ece33700-bib-0040] Paredes, R. , Harding, A. M. A. , Irons, D. B. , Roby, D. D. , Suryan, R. M. , Orban, R. A. , … Kitaysky, A. (2012). Proximity to multiple foraging habitats enhances seabirds’ resilience to local food shortages. Marine Ecological Progress Series, 471, 253–269. https://doi.org/10.3354/meps10034

[ece33700-bib-0041] Paredes, R. , Orben, R. A. , Suryan, R. M. , Irons, D. B. , Roby, D. D. , Harding, A. M. A. , … Kitaysky, A. (2014). Foraging responses of black‐legged kittiwakes to prolonged food‐shortages around colonies on the Bering Sea shelf. PLoS One, 9(3), e92520 https://doi.org/10.1371/journal.pone.0092520 24671108 10.1371/journal.pone.0092520 PMC3966792

[ece33700-bib-0042] Pettex, E. , Lorentsen, S.‐H. , Grémillet, D. , Gimenez, O. , Barrett, R. T. , Pons, J.‐B. , … Bonadonna, F. (2012). Multi‐scale foraging variability in Northern gannet (*Morus bassanus*) fuels potential foraging plasticity. Marine Biology, 159, 2743 https://doi.org/10.1007/s00227-012-2035-1

[ece33700-bib-0043] Piatt, F. P. , Harding, A. M. A. , Shultz, M. , Speckman, S. G. , van Pelt, T. I. , Drew, G. S. , & Kettle, A. B. (2007). Seabirds as indicators of marine food supplies: Cairns revisited. Marine Ecological Progress Series, 352, 221–234. https://doi.org/10.3354/meps07078

[ece33700-bib-0044] Poloczanska, E. S. , Brown, C. J. , Sydeman, W. J. , Kiessling, W. , Schoeman, D. S. , Moore, P. J. , … Richardson, A. J. (2013). Global imprint of climate change on marine life. Nature Climate Change, 3, 919–925. https://doi.org/10.1038/NCLIMATE1958

[ece33700-bib-0045] Ponchon, A. , Grémillet, D. , Christensen‐Dalsgaard, S. , Erikstad, K. E. , Barrett, R. T. , Reiertsen, T. K. , … Boulinier, T. (2014). When things go wrong: Intra‐season dynamics of breeding failure in a seabird. Ecosphere, 5(1), 4 https://doi.org/10.1890/ES13-00233.1

[ece33700-bib-0046] Rey, F. (2004). Phytoplankton: The grass of the sea In Skjoldal H. R. (Ed.), The Norwegian Sea Ecosystem (pp. 97–136). Trondheim, Norway: Tapir Academic Press.

[ece33700-bib-0047] Robertson, G. S. , Bolton, M. , Grecian, W. J. , & Monaghan, P. (2014). Inter‐ and intra‐year variation in foraging areas of breeding kittiwakes (*Rissa tridactyla*). Marine Biology, 161, 1973–1986. https://doi.org/10.1007/s00227-014-2477-8 25170176 10.1007/s00227-014-2477-8 PMC4139585

[ece33700-bib-0048] Sandvik, H. , Barrett, R. T. , Erikstad, K. E. , Myksvoll, M. S. , Vikebø, F. , Yoccoz, N. G. , … Systad, G. H. (2016). Modelled drift patterns of fish larvae link coastal morphology to seabird colony distribution. Nature Communications, 7, 11599 https://doi.org/10.1038/ncomms11599 10.1038/ncomms11599 PMC4869253 27173005

[ece33700-bib-0049] Saraux, C. , Robinson‐Laverick, S. M. , Le Maho, Y. , Ropert‐Coudert, Y. , & Chiaradia, A. (2011). Plasticity in foraging strategies of inshore birds: How Little Penguins maintain body reserves while feeding offspring. Ecology, 92, 1919–1916. https://doi.org/10.1890/11-0407.1 10.1890/11-0407.1 22073782

[ece33700-bib-0050] Schoener, T. W. (1971). Theory of breeding strategies. Annual Review of Ecology and Systematics, 2, 369–404. https://doi.org/10.1146/annurev.es.02.110171.002101

[ece33700-bib-0051] Schulte‐Hostedde, A. I. , Zinner, B. , Millar, J. S. , & Hickling, G. J. (2005). Restitution of mass‐size residuals: Validation body condition indices. Ecology, 86, 155–163. https://doi.org/10.1890/04-0232

[ece33700-bib-0052] Skjoldal, H. R. , Dalpadado, P. , & Dommasnes, A. (2004). Food webs and trophic interactions In Skjoldal H. R. (Ed.), The Norwegian sea ecosystem (pp. 447–506). Trondheim, Norway: Tapir Academic Press.

[ece33700-bib-0053] Staniland, I. J. , Trathan, P. , & Martin, A. R. (2006). Consequences of prey distribution for the foraging behaviour of top predators In Boyd I., Wanless S., & Camphuysen C. J. (Eds.), Top predators in marine ecosystems – Their role in monitoring and management (pp. 131–142). Cambridge, UK: Cambridge University Press https://doi.org/10.1017/CBO9780511541964

[ece33700-bib-0054] Stearns, S. C. (1992). The evolution of life histories. Oxford, UK: Oxford University Press.

[ece33700-bib-0055] Stephens, D. W. (2008). Decision ecology: Foraging and the ecology of animal decision making. Cognitive, Affective, & Behavioral Neuroscience, 8, 475–484. https://doi.org/10.3758/CABN.8.4.475 10.3758/CABN.8.4.475 19033242

[ece33700-bib-0056] Stephens, D. W. , & Krebs, J. R. (1986). Foraging theory. Princeton, NJ: Princeton University Press.

[ece33700-bib-0057] Suryan, R. M. , Irons, D. B. , Kaufman, M. , Benson, J. , Jodice, P. G. R. , Roby, D. D. , … Brown, E. D. (2002). Short‐term fluctuations in forage fish availability and the effect on prey selection and brood‐rearing in the black‐legged kittiwake Rissa tridactyla. Marine Ecology Progress Series, 236, 273–287. https://doi.org/10.3354/meps236273

[ece33700-bib-0058] Thaxter, C. B. , Lascelles, B. , Sugar, K. , Cook, A. S. C. P. , Roos, S. , Bolton, M. , … Burton, N. H. K. (2012). Seabird foraging ranges as a preliminary tool for identifying candidate Marine Protected Areas. Biological Conservation, 156, 53–61. https://doi.org/10.1016/j.biocon.2011.12.009

[ece33700-bib-0059] Tibshirani, R. , Walther, G. , & Hastie, T. (2001). Estimating the number of clusters in a data set via gap statistics. Journal of the Royal Statistical Society: Series B, 63, 411–423. https://doi.org/10.1111/1467-9868.00293

[ece33700-bib-0060] van der Kooij, J. , Scott, B. E. , & Mackinson, S. (2008). The effects of environmental factors on daytime sandeel distribution and abundance on the Dogger Bank. Journal of Sea Research, 60, 201–209. https://doi.org/10.1016/j.seares.2008.07.003

[ece33700-bib-0061] Van Moorter, B. , Visscher, D. R. , Jerde, C. L. , Frair, J. L. , & Merrill, E. H. (2010). Identifying Movement States From Location Data Using Cluster Analysis. Journal of Wildlife Management, 74(3), 588–594. https://doi.org/10.2193/2009-155

[ece33700-bib-0062] Vandenabeele, S. P. , Shepard, E. L. , Grogan, A. , & Wilson, R. P. (2012). When three per cent may not be three per cent; device‐equipped seabirds experience variable flight constraints. Marine Biology, 159, 1–14. https://doi.org/10.1007/s00227-011-1784-6

[ece33700-bib-0063] Vandenabeele, S. P. , Wilson, R. P. , & Grogan, A. (2011). Tags on seabirds: How seriously are instrument‐induced behaviours considered? Animal Welfare, 20, 559–571.

[ece33700-bib-0064] Votier, S. , Bearhop, S. , Witt, M. J. , Inger, R. , Thompson, D. , & Newton, J. (2010). Individual responses of seabirds to commercial fisheries revealed using GPS tracking, stable isotopes and vessel monitoring systems. Journal of Applied Ecology, 47, 487–297. https://doi.org/10.1111/j.1365-2664.2010.01790.x

[ece33700-bib-0065] Wakefield, E. D. , Owen, E. , Baer, J. , Carroll, M. J. , Daunt, F. , Dodd, S. G. , … Bolton, M. (2017). Breeding density, fine‐scale tracking, and large‐scale modelling reveal the regional distribution of four seabird species. Ecological Applications, 27(7), 2074–2091. https://doi.org/10.1002/eap.1591 28653410 10.1002/eap.1591

[ece33700-bib-0067] Weimerskirch, H. (1998). How can a pelagic seabird provision its chick when relying on a distant food resource? Cyclic attendance at the colony, foraging decision and body condition in Sooty shearwaters. Journal of Animal Ecology, 67, 99–109. https://doi.org/10.1046/j.1365-2656.1998.00180.x

[ece33700-bib-0068] Weimerskirch, H. (2007). Are seabirds foraging for unpredictable resources. Deep Sea Research Part II Topical Studies in Oceanography, 54, 211–223. https://doi.org/10.1016/j.dsr2.2006.11.013

[ece33700-bib-0069] Weimerskirch, H. , Chastel, O. , Ackermann, L. , Chaurand, T. , Cuenotchaillet, F. , Hindermeyer, X. , & Judas, J. (1994). Alternate long and short foraging trips in pelagic seabird parents. Animal Behaviour, 47, 472–476. https://doi.org/10.1006/anbe.1994.1065

[ece33700-bib-0070] Weimerskirch, H. , Le Corre, M. , Ropert‐Coudert, Y. , Kato, A. , & Marsac, F. (2006). Sex‐specific foraging behaviour in a seabird with reversed sexual dimorphism: The red‐footed booby. Oecologia, 146, 681–691. https://doi.org/10.1007/s00442-005-0226-x 16195880 10.1007/s00442-005-0226-x

[ece33700-bib-0071] Welcker, J. , Beidersdorf, A. , Varpe, Ø. , & Steen, H. (2012). Mass fluctuations suggest different functions of bimodal foraging trips in a central‐place forager. Behavioral Ecology, 23(6), 1372–1378. https://doi.org/10.1093/beheco/ars131

[ece33700-bib-0072] Yamamoto, T. , Kokubun, N. , Kikuchi, D. M. , Sato, N. , Takahashi, A. , Will, A. P. , … Watanuki, Y. (2016). Differential responses of seabirds to environmental variability over 2 years in the continental shelf and oceanic habitats of southeastern Bering Sea. Biogeosciences, 13, 2405–2414. https://doi.org/10.5194/bg-13-2405-2016

[ece33700-bib-0073] Ydenberg, R. C. , & Davies, W. E. (2010). Resource geometry and provisioning routines. Behavioural Ecology, 21, 1170–1178. https://doi.org/10.1093/beheco/arq127

[ece33700-bib-0074] Zuur, A. F. , Ieno, E. N. , Walker, N. , Saveliev, A. A. , & Smith, G. M. (2009). Mixed effects models and extensions in ecology with R. New York, NY: Springer https://doi.org/10.1007/978-0-387-87458-6

