# Supplementary material for: Taking a trip to the shelf: Behavioral decisions are mediated by the proximity to foraging habitats in the black‐legged kittiwake
Source: Ecol Evol. 2017 Dec 10;8(2):866–78. doi: 10.1002/ece3.3700 (PMC5773323; doi:10.1002/ece3.3700)
Supplement: Supplementary file 1 [file ECE3-8-866-s001.docx]

'The following Supporting Information is available for this article online'

Taking a trip to the shelf: behavioural decisions are mediated by the proximity to foraging habitats in the black-legged kittiwake

Short title: Foraging decisions of black-legged kittiwakes

Signe Christensen-Dalsgaard*^a,b^, Roel May^b^, Svein-Håkon Lorentsen^b^

^a^ Department of Biology, Norwegian University of Science and

Technology (NTNU), 7491 Trondheim, Norway,

^b^ Norwegian Institute for Nature Research (NINA), P.O. Box 5685 Torgard, 7034 Trondheim, Norway,

* Corresponding author: signe.dalsgaard@nina.no

**Appendix S1: Deriving behavioural states from high-resolution GPS-locations**

**Data analysis**

GPS-data points (geographical positions hereafter locations) taken approximately every 2 minutes were used for the analysis. When the GPS-loggers were programmed to record a position every minute, every second position was used for the analysis. As the time to acquire GPS fixes may vary due to the location and orientation of the tag, locations were not always exactly 2 minutes apart, but no post-processing was applied to the data to account for this. A maximum speed of 80 km h^-1^ was used to filter out locations, as they were likely to have been caused by locational error (following Paredes et al. 2012). GPS locations from birds sitting on the nest (and a buffer of 100 meters around the nest to account for potential inaccuracy of locations) were removed. All the remaining locations were processed to calculate speed, change in speed and the absolute turning angles between sequential pairs of locations of the individual trajectories using the R-package adehabitat (Calenge 2006). All variables were log-transformed to reduce positive skew and range standardization following Steinley (2006) was applied (Van Moorter et al. 2010). Due to the loss of satellite reception, some locations were not recorded, leading to data gaps. As these may lead to erroneous calculations of the movement variables, the last two data points before each gap were identified using the function check.holes and omitted from the analysis to enable correct calculation of speed, change in speed and turning angles (in total 7766 points were removed, leaving 212 571 locations from foraging trips).

To identify the number of clusters in the dataset we applied gap-statistics, where the output of a clustering algorithm, was compared to the within-cluster dispersion with the appropriate reference null distribution (Tibshirani, Walther & Hastie 2001). The gap statistics, using a *k*-means clustering was carried out using the function index.GAP (Van Moorter et al. 2010, script available at <http://ase-research.org/moorter/>), using the Hartigan-Wong *k*-means clustering algorithm (similar results were obtained using the Loyd algorithm), with the variables being speed, change in speed and absolute turning angles.

To see how the different clusters were separated and structured, a classification tree was created using the R package “tree” (Ripley 2016), where the tree is grown by binary recursive partitioning using the response in the specified formula and choosing splits from the terms of the right-hand-side. The split which maximizes the reduction in impurity is chosen, the data set split and the process repeated (Ripley 1996).

VALIDATION OF CLUSTERING USING SALTWATER IMMERSION

Information on saltwater immersion was used to evaluate the quality of the identification of groups based on the *k*-means clustering.

Data from the 21 TDR-loggers (wet/dry values every second) from kittiwakes breeding on Anda in 2013, were coupled with geographical locations from the GPS-loggers, creating an average value of saltwater immersion for each 2-minute interval between GPS-points. The prediction that TDR-loggers from commuting, foraging and resting birds show low, varying and high saltwater immersion, respectively, was tested. As kittiwakes can rest both on water and on land, locations of birds resting on land can give erroneous low values of immersion for the resting phase. All GPS-locations from terrestrial areas (land or the intertidal zone covering the zone between high and low tide), a total of 10 117 (72.2%) of the 14 039 locations in the behavioural cluster “resting”, were therefore removed from the dataset before analysing the wet/dry status of the different behavioural states.

Data from GLS-loggers were collected on Anda in 2013 and 2014. The GLS-loggers used, record the wet status every 3 second. These are summed for 10-minute intervals so that a value of 200 indicate that the loggers has been submerged for the whole period, and a value of 0 that it has been dry all the time. To test for differences in saltwater immersion using the 10-minute interval registered by GLS-loggers (hereafter named “GLS-period”), the information on which cluster each GPS-point was assigned to, was summed to create a proportion of the respective clusters for every GLS-period. To account for the possibility of some of the GLS-periods being affected by gaps in the data, only GLS-periods where three or more GPS-points were registered were included in the analysis. In addition, to account for kittiwakes resting on land, the proportion of the locations being on land based on information from the GPS-logger was calculated, and GLS-periods where more than 10% of the locations were on land were excluded (in total 10.4% of the GLS-periods). The saltwater immersion per period was analysed for each of the clusters using a quasi-binomial generalized linear model (GLM) where number of 3 second periods that were wet was the response variable and the total number of periods in the time block (10 mins) were the binomial denominator. Additionally, to assess how the different clusters were distributed in the different categories, the GLS-periods were separated in three immersion categories: sustained flight (≥ 95% dry, hereafter “commuting”), sitting on the water (≥ 95% wet, hereafter “resting”) and foraging behaviour (> 5% dry and > 5% wet, representing a succession of short flights while searching for prey and short wet bouts of sitting on the water, hereafter “foraging”).

MODEL VALIDATION OF K-MEANS CLUSTERING USING CROSS VALIDATION

To measure the predictability and stability of the *k*-means cluster assignment, three different cross-validations were conducted using the function kmeans.CV. First, the source data was binned into ten equal-sized random groups. Nine groups were selected to train the k-means clustering model upon which the cluster assignment for the tenth group was predicted using Mahalanobis multivariate distance. This process was iterated for each random group. Simultaneously, the data was split into k equal-sized distance groups based on Sturges’ formula for the standardized multivariate distance from each cluster’s centroid. Secondly, to evaluate the effect of data quantity on the cluster’s stability an additive validation was performed by iteratively adding one additional random group to train the k-means clustering model to predict cluster assignment for the remaining groups. The third cross-validation was conducted with a temporal and spatial partitioning of the data by year and colony, to measure the temporal and spatial predictability of the k-means model. Each of the unique groups’ cluster assignments was predicted based on the remaining groups. The probability of successful assignment for each of the k-means clusters were plotted against distance from the cluster centroid and cross-validation performance was evaluated. Potential grouping effects on successful cluster assignment was evaluated using a likelihood-ratio test including a random grouping of bird ID. The area under the curve (AUC) was calculated for the plotted results. The mean R^2^ of between-groups sum-of-squares to the total sum-of-squares was calculated for each of the cross-validations. Finally, the cross-validation performance is summarized in a contingency table.

To evaluate the cluster assignment error occurring along a bird’s trajectory, each location’s cluster assignment was compared to a sequence of n preceding and subsequent locations; whereby n was increased from two to ten on either side of each evaluated location. When all surrounding locations were assigned to one other cluster this signified a cluster assignment error. The proportional assignment error was plotted for each cluster and sequence length. The point of inflection was used to select what number of preceding and subsequent locations to use. The assignment errors among clusters for the inflection point were summarized in a contingency table.

Statistical analysis was carried out using R, version 3.2.3 (R Development Core Team, 2008). All specifically developed functions can be provided.

**Results**

The results from the gap-statistics for different numbers of clusters showed that there was statistically significant support for three clusters of movement behaviour (Fig. A1.1), suggesting that the at-sea behaviour of kittiwakes was best described as three distinct behavioural states.


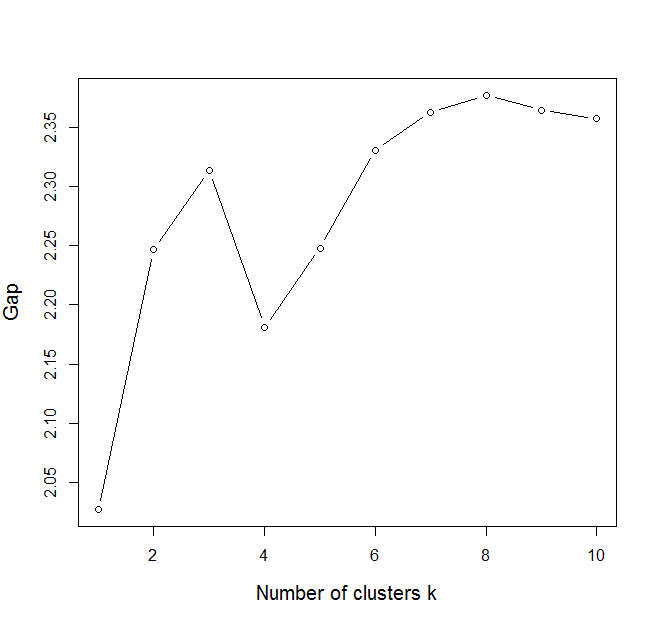


**Fig. A1.1.** Gap statistics for different numbers of clusters with standard error for 1 – 10 clusters.

The first cluster was characterized by intermediate speed (median speed 5.8 ms^-1^) with large changes in speed and a relative high tortuosity in the track suggesting a searching or foraging behaviour (hereafter referred to as “foraging”, Fig. A1.2). The second cluster was defined by a fast movement trajectory (median speed 9.9 ms^-1^) with little change in speed and comparatively straight tracks (Fig. A1.2), corresponding to a commuting behaviour (hereafter referred to as “commuting”). The third cluster was characterized by the birds primarily flying at slow speed (median speed 0.3 ms^-1^), little change in speed and with highly tortuous tracks, which was interpreted as a resting, with the birds primarily being passively displaced by currents and waves (hereafter referred to as “resting”, Fig. A1.2).


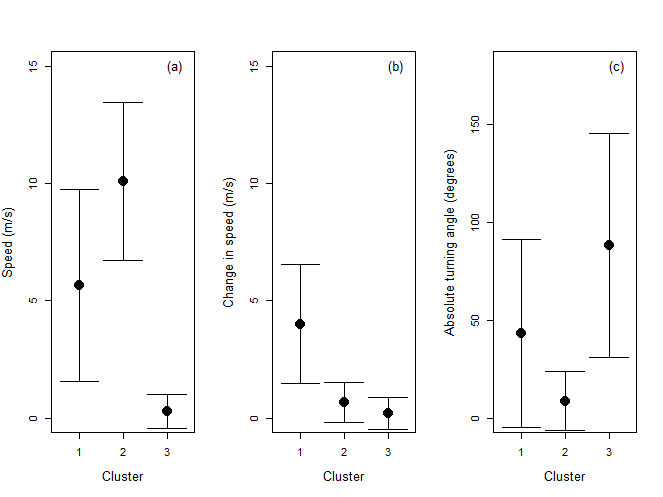


**Fig. A1.2.** Statistical description of behavioural clusters inferred from kittiwake locations during foraging trips. Median value ± SD is shown for a) speed, b) change in speed and c) absolute turning angle. The interpretation of clusters is, 1: foraging 2: commuting behaviour, and 3: resting behaviour.

The classification tree showed that the initial separation of data was based on speed, with speeds below 1.49 ms^-1^ being mainly the behavioural cluster “resting”. Speeds higher than 1.49 ms^-1^ were classified as either “foraging” or commuting, with the change in speed being the main difference between these two (separation value of 1.55 ms^-1^). The turning angle differentiated between “foraging” and “commuting”, with commuting having less turning angle.

From a visual inspection of the cartographic distribution of GPS-locations of the individual bird tracks with the clustering classified by the *k*-means, it is apparent that the patterns of behavioural states assigned to the points seem to be consistent with the patterns of GPS-locations (Fig. A1.3). The behavioural state “foraging” is mainly more densely distributed over smaller areas where the bird has spent more time. “Commuting” is seen as primarily long stretches of directional flight between colony and apparent feeding areas and between apparent feeding areas, and “resting” as periods of passive floating with currents. Though having up to a 5-fold difference in trip length the assignment of behavioural clusters appeared consistent between trajectories from the two colonies studied.

**Fig. A1.3.** Example of individual movement trajectories where behavioural states have been classified, from Anda (left) and Sør-Gjæslingan (right). Locations assigned cluster 1 (foraging), 2 (commuting) and 3 (resting) are indicated with respectively yellow, green, and red points, respectively. The position of the colony is marked with a star.

VALIDATION USING SALTWATER IMMERSION

The results from the GLS-loggers showed that the saltwater immersion significantly decreased with increased proportion of “commuting” and “foraging” (respectively t = -52.8, P < 0.001 and t = -6.8, P < 0.001) and increased significantly with increased proportion of “resting” (t=60.7, P<0.001)

When separating in the three immersion categories dry, intermediate and wet (respectively ≤ 5%, 5-95% and ≥ 95% immersion), the different clusters were clearly distributed (Fig. A1.4). In the category “dry” the behavioural cluster “commuting” was dominating with a median value of 0.77 compared to 0.17 and 0 respectively for the behavioural cluster “foraging” and “resting”. As opposed to this, in the category “wet” the behavioural cluster “resting” had a median value of 1 and the two other clusters both 0.


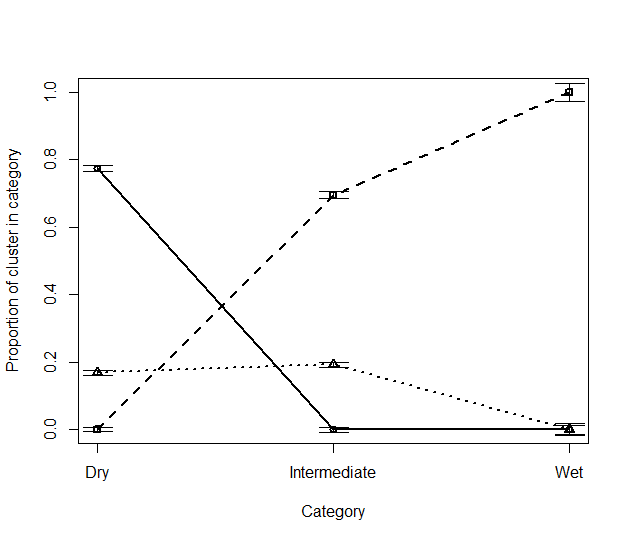


**Fig. A1.4.** Median and confidence interval of the proportion of clusters in the different categories of immersion based on information from GLS-loggers. Full line represent cluster “commuting”, dotted line represents the cluster “foraging” and striped line the cluster “resting”.

When the immersion data from TDR-loggers were coupled with locational points in the three different clusters identified, the difference in activity was also apparent. The first cluster (“foraging”) had low saltwater immersion (median proportion of time immersed = 0.04, Table A1.1). The second cluster (“commuting”) also had very low saltwater immersion (median proportion of time immersed = 0.00, Table A1.1), whereas the third cluster (“resting”) had a very high proportion of time immersed (median proportion of time immersed = 0.93, Table A1.1).

**Table A1.1.** Relative distribution of saltwater immersion in % for the three clusters based on data from TDR-loggers, ranging from 0% wet to 100% wet. Left in the table are the clusters identified through the k-means clustering and on the right-side clusters after carrying out correction for cluster assignment error.

|  | Immersion *k*-mean clusters | | | |  |  | Immersion, corrected for assignment error | | | |
| --- | --- | --- | --- | --- | --- | --- | --- | --- | --- | --- |
| **Cluster** | **n** | **mean** | **se** | **median** |  |  | **n** | **mean** | **se** | **median** |
| **foraging** | 4679 | 28.83 | 0.54 | 3.25 |  |  | 4542 | 28.36 | 0.55 | 2.33 |
| **commuting** | 4657 | 8.60 | 0.36 | 0 |  |  | 4619 | 8.27 | 0.36 | 0 |
| **resting** | 6649 | 72.63 | 0.44 | 93.42 |  |  | 6824 | 71.91 | 0.43 | 92.24 |

VALIDATION OF CLUSTERING METHOD

Results from the random cross-validation showed that for both the 10-fold cross-validation and the cumulative approach the predictions by the *k*-means model were very stable. For both approaches there were no significant differences between observed and predicted values (for cross-validation 1 and 2, p = 0.18 and p = 0.66 respectively) and the AUC was 0.95 for both cross-validations. The mean R^2^ was 0.53 (SD = 0.002).

The analysis of the effect of distance from cluster centroid on the probability of successful assignment showed that there were differences in the effect of distance between the different clusters (p < 0.001). For both the clusters described as “resting” and “commuting” there was a pattern of the cross-validation probability decreasing with increased distance from cluster centroid, most markedly for the latter. As opposed to this, the cluster “foraging” was stable from centre to the periphery. This was also reflected in the overall cross-validation probability which was highest for cluster 2 (Fig. A1.5). It should however be noted that with an AUC of 0.94, there was still an overall good fit in the analysis of the effect of distance from centroid.

For the cross-validation conducted with data partitioned into groups by year and colony the overall predictive power was very good, with an AUC of 0.94 (Fig. A1.5). There was a significant difference between predicted and observed values (p < 0.001), this can however be assigned to the very large sample size. The cross-validation probability varied between 0.93 ± 0.002 (mean ± SD) and 0.96 ± 0.001 depending on which colonies and years were used as training sets (Fig. A1.5). The mean R^2^ of all the groups in the cross-validations was 0.53 (SD = 0.002).


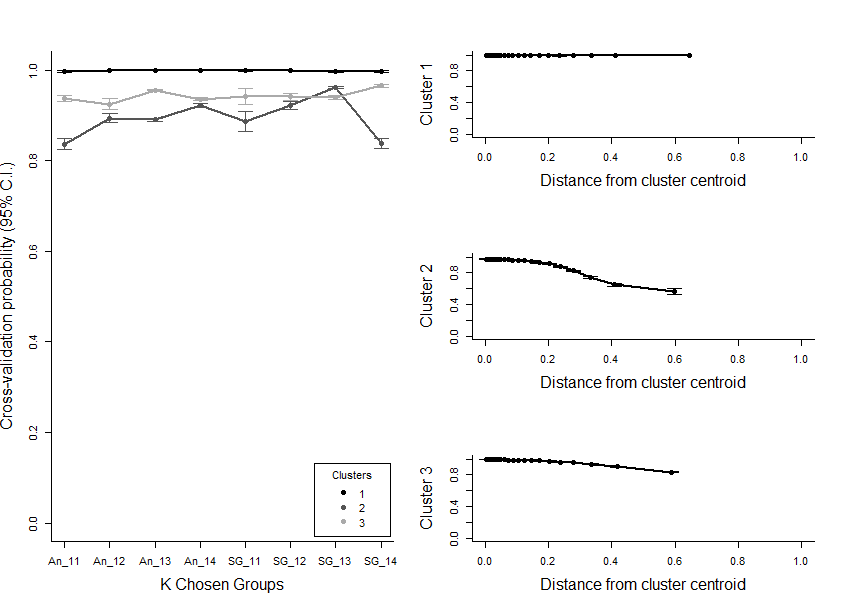


Fig. A1.5. Cross-validation probability for the groups of year and colony (left), the x-axis is colony years (e.g. An_11 being Anda in 2011, and SG_11 being Sør-Gjæslingan in 2011). To the right, the probability of successful cluster assignment across clusters plotted against equal-sized binned distance groups ranging from cluster centre (at cluster centroid) to cluster periphery (farthest away from cluster centroid). ). Cluster 1 is “foraging”, cluster 2 is “commuting” and cluster 3 is “resting”.

**Table A1.2.** Contingency table for k-fold cross validation using with a temporal and spatial partitioning of the data, percentages are included in the parentheses. The number of observations correctly classified per behaviour is shown in bold.

|  | Predicted | | | | |
| --- | --- | --- | --- | --- | --- |
| Observed |  | Foraging | Commuting | Resting | Sum |
|  | Foraging | 60013 **(99.9)** | 25 (0.0) | 62 (0.0) | 60100 |
|  | Commuting | 6634 (8.4) | 71645 **(91.1)** | 373 (0.5) | 78652 |
|  | Resting | 3741 (4.8) | 310 (0.4) | **73515 (94. 8)** | 77566 |
|  | Sum | 70388 | 71980 | 73950 |  |

When comparing the clustering identified in the original k-means analysis (observed values) with the ones predicted through the k-fold validation, the behavioural state “foraging” was most often predicted correctly with 99.9% of the observed values being predicted correctly (Table A1.2). For the behavioural state “commuting”, 91.1% were assigned to the right cluster with 8.4% being assigned falsely to “foraging”. Similarly, for the behavioural state “resting” 94.8% were predicted correctly with 4.8% of the values falsely being assigned to “foraging”.

EVALUATION OF CLUSTER ASSIGMENT ERROR

The evaluation of error in cluster assignment occurring along the trajectory where surrounding locations were assigned to one other cluster, showed that for “resting” and “commuting” there was a low error (Fig. A1.6). The inflection point for all three clusters was at 2, and when using this point, where two or more points on each side were used, the proportional error approached 7.5 for foraging, 3% for commuting and 1% for resting.


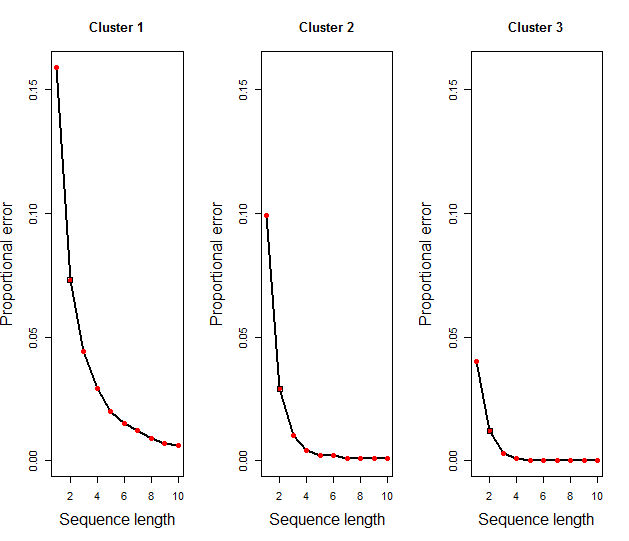


**Fig. A1.6.** Proportional error in cluster assignment along the trajectory. Each location’s cluster assignment is compared to 1 – 10 preceding and subsequent locations. The inflection point is marked with a square. Cluster 1 is “foraging”, cluster 2 is “commuting” and cluster 3 is “resting”.

When summarizing the information using the inflection point of two locations on each side of the point (Table A1.3), this pattern was also reflected with “foraging” having 4375 erroneous points in assignment, “commuting” having 2264 points and “resting” having 894 points being changed.

**Table A1.3**. Contingency table of assignment errors among clusters using two locations on each side of the point, percentages of total number in the respective clusters are included in the parentheses.

|  | Foraging | Commuting | Resting |
| --- | --- | --- | --- |
| Foraging | 0 | 2657 (4.4) | 1718 (2.9) |
| Commuting | 1908 (2.4) | 0 | 356 (0.5) |
| Resting | 845 (1.1) | 49 (0.0) | 0 |

To assess what effect the assignment error had, the locations where the two surrounding locations were assigned to one other cluster, were substituted with this cluster number. This data was used to re-analyse the immersion data from the TDR-loggers to get the immersion characteristics for the respective clusters. There were not any apparent differences in the immersion between the two datasets (Table A1.3).

**Discussion**

The results from the gap analysis showed that there was most support for assigning the locational data to three different behavioural clusters. Based on the speed, change in speed and tortuosity the three behavioural clusters identified through the k-means analysis could be interpreted as commuting (high speed, little change in speed and low tortuosity), resting (low speed, little change in speed and high tortuosity) and foraging for food (relatively high speed, large change in speed and high tortuosity). This categorization is similar to that used in other studies (e.g. Dean et al. 2012, Paredes et al. 2012). In this study, we tested the methods suitability to cluster data by using three movement variables that can easily be derived from movement trajectories. The method is, however, flexible with regard to the number of variables to include, and if available and suitable in answering the specific research questions one could additionally have included e.g. flight height, acceleration or immersion in water.

The data from the GLS-loggers showed that for each of the clusters identified, there was a significant effect of the proportion of the cluster on the degree of saltwater immersion. Using high-resolution immersion data from the TDR-loggers this pattern was substantiated, with one cluster having very high rates of immersion, one having almost no immersion and one having low immersion rates, but with large variability, respectively interpreted as resting behaviour, commuting behaviour and a foraging behaviour. These patterns correspond well with the movement patterns of the respective clusters, giving a strong indication of the quality of the cluster assignment.

Overall, the k-means clustering method did prove to be very robust when tested using cross-validation analysis. There were, however, some between-cluster differences in cross-validation probability, with the behavioural cluster “foraging” having highest values. This tendency was also reflected in the performance of the model when looking at the distance of points from cluster centroid, where the cluster “foraging” performed better than the two other clusters. This result seems somewhat contradictory to what anticipated, as one could have expected the apparently more discreet and directional behavioural states of resting and commuting to be more robust to predictions. An explanation can possibly be found in the fact that the behavioural states are not inherently discreet though we attempt to place them in clusters. In this clustering the two extreme movement clusters of “resting” and “commuting” are made to include some of the “foraging” (e.g. respectively small-scale or large-scale searching), making the “foraging” cluster more conservative. Indeed, the classification tree did indicate that there were some locations of cluster “foraging” that fell into the overall branch of “resting” and it is likely that it is these locations that are being shifted to “foraging” in the cross-validation.

Our results indicate that the overall predictive power was very good, indicating that data from different colonies could be included in the same analysis as long as the sampling interval is similar.

**Literature cited**

Calenge, C. (2006) The package adehabitat for the R software: a tool for the analysis of space and habitat use by animals. Ecological Modelling, 197, 516-519.

Dean, B., Freeman, R., Kirk, H., Leonard, K., Phillips, R.A., Perrins, C.M. & Guilford, T. (2012) Behavioural mapping of a pelagic seabird: combining multiple sensors and a hidden Markov model reveals the distribution of at-sea behavior. Journal of The Royal Society Interface, http://dx.doi.org/10.1098/rsif.2012.0570

Paredes, R., Harding A.M.A., Irons, D.B., Roby, D.D., Suryan, R.M., Orban, R.A., Renner, H., Young, R. & Kitaysky, A. (2012) Proximity to multiple foraging habitats enhances seabirds’ resilience to local food shortages. Marine Ecology Progress Series 471: 253 – 269.

R Development Core Team, 2008. R: A Language and Environment for Statistical Computing. R foundation for Statistical Computing. Vienna, Austria.

Brian Ripley (2016) tree: Classification and Regression Trees. R package version 1.0-37. <https://CRAN.R-project.org/package=tree>

Ripley, B. D. 1996. Pattern Recognition and Neural Networks. Cambridge University Press, Cambridge. Chapter 7.

Tibshirani, R., Walther, G. & Hastie, T. 2001. Estimating the number of clusters in a data set via gap statistics. J. R. Statist. Soc. B. 63:411-423

Van Moorter, B., Visscher, D.R., Jerde, C.L., Frair, J.L. & Merrill, E.H. (2010) Identifying Movement States From Location Data Using Cluster Analysis. Journal of Wildlife Management, 74(3), 588-594.

**Appendix S2: Prevailing wind directions during foraging trips at the two study sites**


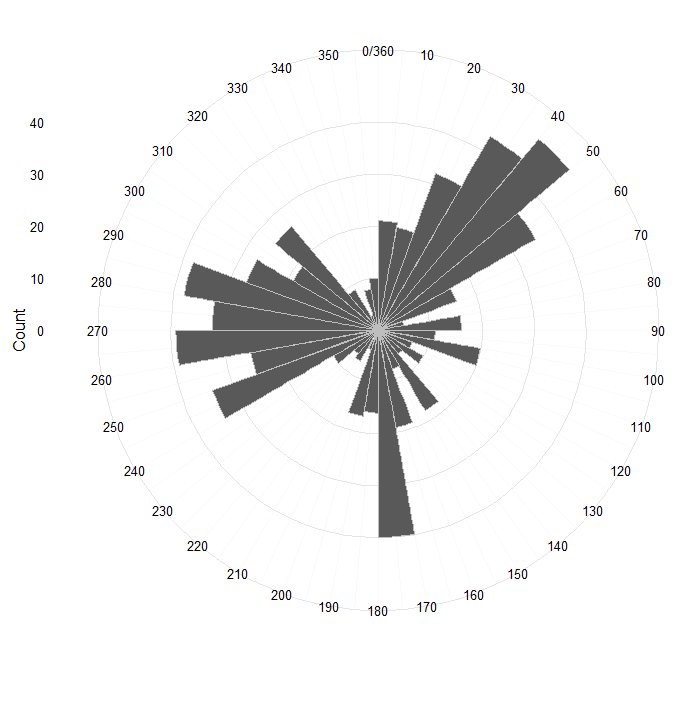


**Fig. S2.1.** Counts of foraging trips of kittiwakes from Anda separated by prevailing wind direction when the trips started. The length of the bars in each figure depicts the total number of trips conducted for each 10° groupings of wind direction.


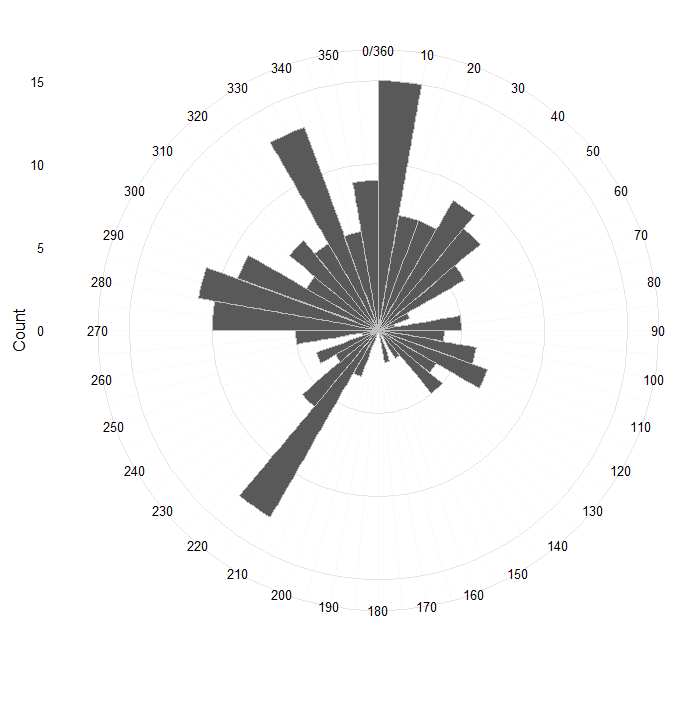


**Fig. S2.2.** Counts of foraging trips of kittiwakes from Sør-Gjæslingan separated by prevailing wind direction when the trips started. The length of the bars in each figure depicts the total number of trips conducted for each 10° groupings of wind direction.

**Appendix S3: Model selection**

**Table S3.1.** Generalised linear mixed models testing for relationships between behaviour during feeding trips from Anda (commuting, foraging and resting) and predictor variables, for respectively oceanic and fjord trips. The model with the lowest AIC is shown in bold.

|  |  |  |  | Oceanic habitat | | |  | Fjord habitat | | |
| --- | --- | --- | --- | --- | --- | --- | --- | --- | --- | --- |
|  |  |  | Df | AICc | ΔAICc | weight |  | AICc | ΔAICc | weight |
| Commute | extrinsic (time) | Depart time | 4 | 2252.71 | 36.10 | 0 |  | 4263.01 | 6.27 | 0.028 |
|  | extrinsic - time | Proportion ebb | 4 | 2248.82 | 32.21 | 0 |  | 4267.39 | 10.65 | 0.003 |
|  | extrinsic (time) + extrinsic (weather) | Depart time*Wind speed | 6 | 2229.22 | 12.61 | 0.002 |  | 4262.80 | 6.06 | 0.031 |
|  | extrinsic (weather) | Wind direction | 5 | 2261.85 | 45.24 | 0 |  | 4259.28 | 2.54 | 0.179 |
|  | extrinsic - weather | Wind speed | 4 | 2247.01 | 30.40 | 0 |  | 4262.94 | 6.20 | 0.029 |
|  | extrinsic - weather | Wind speed*Wind direction | 8 | 2234.66 | 18.05 | 0 |  | 4261.52 | 4.78 | 0.059 |
|  | intrinsic + extrinsic (weather) | Wind speed*Chick age | 6 | 2249.81 | 33.20 | 0 |  | 4265.05 | 8.31 | 0.01 |
|  | intrinsic + extrinsic (weather) | BCI*Wind speed | 6 | 2250.29 | 33.68 | 0 |  | 4269.26 | 12.52 | 0.001 |
|  | intrinsic | Sex | 4 | 2261.77 | 45.16 | 0 |  | 4268.94 | 12.20 | 0.001 |
|  | intrinsic | Chick age | 4 | 2261.82 | 45.21 | 0 |  | 4268.53 | 11.79 | 0.002 |
|  | intrinsic | BCI | 4 | 2262.08 | 45.47 | 0 |  | 4268.35 | 11.61 | 0.002 |
|  | intrinsic | BCI*Sex | 6 | 2265.81 | 49.20 | 0 |  | 4264.28 | 7.54 | 0.015 |
|  | intrinsic + extrinsic (time) | Depart time*Chick age | **6** | **2216.61** | **0.00** | **0.998** |  | **4256.74** | **0.00** | **0.638** |
|  | intercept | 0-model | 3 | 2260.14 | 43.53 | 0 |  | 4267.56 | 10.82 | 0.003 |
| Forage | extrinsic (time) | Depart time | 4 | 1768.41 | 10.40 | 0.003 |  | 3733.63 | 37.27 | 0 |
|  | extrinsic - time | Proportion ebb | 4 | 1771.17 | 13.16 | 0.001 |  | 3732.27 | 35.91 | 0 |
|  | extrinsic (time) + extrinsic (weather) | Depart time*Wind speed | 6 | **1758.01** | **0.00** | **0.614** |  | 3705.86 | 9.50 | 0.008 |
|  | extrinsic (weather) | Wind direction | 5 | 1779.03 | 21.02 | 0 |  | 3712.76 | 16.40 | 0 |
|  | extrinsic - weather | Wind speed | 4 | 1777.53 | 19.52 | 0 |  | 3705.30 | 8.94 | 0.011 |
|  | extrinsic - weather | Wind speed*Wind direction | 8 | 1762.05 | 4.04 | 0.081 |  | 3703.24 | 6.88 | 0.03 |
|  | intrinsic + extrinsic (weather) | Wind speed*Chick age | 6 | 1769.27 | 11.26 | 0.002 |  | **3696.36** | **0.00** | **0.939** |
|  | intrisic + extrinsic (weather) | BCI*Wind speed | 6 | 1779.68 | 21.67 | 0 |  | 3729.40 | 33.04 | 0 |
|  | intrinsic | Sex | 4 | 1781.75 | 23.74 | 0 |  | 3727.19 | 30.83 | 0 |
|  | intrinsic | Chick age | 4 | 1773.83 | 15.82 | 0 |  | 3724.23 | 27.87 | 0 |
|  | intrinsic | BCI | 4 | 1781.70 | 23.69 | 0 |  | 3731.84 | 35.48 | 0 |
|  | intrinsic | BCI*Sex | 6 | 1783.19 | 25.18 | 0 |  | 3705.18 | 8.82 | 0.011 |
|  | intrinsic + extrinsic (time) | Depart time*Chick age | 6 | 1759.45 | 1.44 | 0.298 |  | 3716.00 | 19.64 | 0 |
|  | intercept | 0-model | 3 | 1780.44 | 22.43 | 0 |  | 3731.60 | 35.24 | 0 |
| Rest | extrinsic (time) | Depart time | 4 | 2357.84 | 22.91 | 0 |  | 4976.07 | 32.04 | 0 |
|  | extrinsic - time | Proportion ebb | 4 | 2446.82 | 111.89 | 0 |  | 4976.29 | 32.26 | 0 |
|  | extrinsic (time) + extrinsic (weather) | Depart time*Wind speed | 6 | 2351.95 | 17.02 | 0 |  | 4964.79 | 20.76 | 0 |
|  | extrinsic (weather) | Wind direction | 5 | 2412.08 | 77.15 | 0 |  | 4964.62 | 20.59 | 0 |
|  | extrinsic - weather | Wind speed | 4 | 2440.78 | 105.85 | 0 |  | 4972.58 | 28.55 | 0 |
|  | extrinsic - weather | Wind speed*Wind direction | 8 | 2382.44 | 47.51 | 0 |  | 4967.76 | 23.73 | 0 |
|  | intrinsic + extrinsic (weather) | Wind speed*Chick age | 6 | 2442.57 | 107.64 | 0 |  | 4966.11 | 22.08 | 0 |
|  | intrisic + extrinsic (weather) | BCI*Wind speed | 6 | 2443.67 | 108.74 | 0 |  | 4977.30 | 33.27 | 0 |
|  | intrinsic | Sex | 4 | 2443.41 | 108.48 | 0 |  | 4975.09 | 31.06 | 0 |
|  | intrinsic | Chick age | 4 | 2446.00 | 111.07 | 0 |  | 4975.11 | 31.08 | 0 |
|  | intrinsic | BCI | 4 | 2445.76 | 110.83 | 0 |  | 4981.19 | 37.16 | 0 |
|  | intrinsic | BCI*Sex | 6 | 2445.48 | 110.55 | 0 |  | 4971.67 | 27.64 | 0 |
|  | intrinsic + extrinsic (time) | Depart time*Chick age | 6 | **2334.93** | **0** | **1** |  | **4944.03** | **0.00** | **1** |
|  | intercept | 0-model | 3 | 2445.24 | 110.31 | 0 |  | 4979.17 | 35.14 | 0 |

**Table S3.1.** Generalised linear mixed models testing for relationships between behaviour during feeding trips from Sør-Gjæslingan (commuting, foraging and resting) and predictor variables, for respectively oceanic and fjord trips. The model with the lowest AIC is shown in bold.

|  |  |  |  | Oceanic habitat | | |  | Fjord habitat | | |
| --- | --- | --- | --- | --- | --- | --- | --- | --- | --- | --- |
|  |  |  | df | AICc | dAICc | w |  | AICc | dAICc | w |
| Commute | extrinsic (time) | Depart time | 4 | 308.98 | 14.86 | 0 |  | 1590.38 | 23.01 | 0 |
|  | extrinsic - time | Proportion ebb | 4 | 307.47 | 13.35 | 0.001 |  | 1590.66 | 23.28 | 0 |
|  | extrinsic (time) + extrinsic (weather) | Depart time*Wind speed | 6 | 303.17 | 9.05 | 0.009 |  | 1590.05 | 22.67 | 0 |
|  | extrinsic (weather) | Wind direction | 5 | 310.88 | 16.76 | 0 |  | 1570.70 | 3.32 | 0.158 |
|  | extrinsic - weather | Wind speed | 4 | 297.48 | 3.36 | 0.153 |  | 1586.14 | 18.76 | 0 |
|  | extrinsic - weather | Wind speed*Wind direction | 8 | 305.88 | 11.76 | 0.002 |  | 1567.38 | 0.00 | 0.831 |
|  | intrinsic + extrinsic (weather) | Wind speed*Chick age | 6 | 303.08 | 8.96 | 0.009 |  | 1576.16 | 8.78 | 0.01 |
|  | intrinsic + extrinsic (weather) | BCI*Wind speed | 6 | 294.12 | 0.00 | 0.818 |  | 1588.82 | 21.44 | 0 |
|  | intrinsic | Sex | 4 | 308.73 | 14.60 | 0.001 |  | 1587.86 | 20.48 | 0 |
|  | intrinsic | Chick age | 4 | 308.47 | 14.35 | 0.001 |  | 1590.68 | 23.30 | 0 |
|  | intrinsic | BCI | 4 | 304.44 | 10.32 | 0.005 |  | 1590.01 | 22.63 | 0 |
|  | intrinsic | BCI*Sex | 6 | 309.68 | 15.56 | 0 |  | 1590.78 | 23.41 | 0 |
|  | intrinsic + extrinsic (time) | Depart time*Chick age | 6 | 313.52 | 19.40 | 0 |  | 1584.54 | 17.16 | 0 |
|  | intercept | 0-model | 3 | 306.90 | 12.77 | 0.001 |  | 1588.57 | 21.19 | 0 |
| Forage | extrinsic (time) | Depart time | 4 | 257.45 | 3.29 | 0.069 |  | 1640.79 | 26.73 | 0 |
|  | extrinsic - time | Proportion ebb | 4 | 257.76 | 3.59 | 0.06 |  | 1692.97 | 78.91 | 0 |
|  | extrinsic (time) + extrinsic (weather) | Depart time*Wind speed | 6 | 260.03 | 5.86 | 0.019 |  | 1636.50 | 22.44 | 0 |
|  | extrinsic (weather) | Wind direction | 5 | 260.84 | 6.67 | 0.013 |  | 1665.57 | 51.51 | 0 |
|  | extrinsic - weather | Wind speed | 4 | 254.17 | 0.00 | 0.358 |  | 1695.09 | 81.03 | 0 |
|  | extrinsic - weather | Wind speed*Wind direction | 8 | 267.24 | 13.07 | 0.001 |  | 1649.10 | 35.04 | 0 |
|  | intrinsic + extrinsic (weather) | Wind speed*Chick age | 6 | 260.45 | 6.28 | 0.016 |  | 1676.48 | 62.42 | 0 |
|  | intrinsic + extrinsic (weather) | BCI*Wind speed | 6 | 260.60 | 6.43 | 0.014 |  | 1698.17 | 84.11 | 0 |
|  | intrinsic | Sex | 4 | 257.14 | 2.97 | 0.081 |  | 1695.26 | 81.20 | 0 |
|  | intrinsic | Chick age | 4 | 257.78 | 3.61 | 0.059 |  | 1694.95 | 80.89 | 0 |
|  | intrinsic | BCI | 4 | 257.79 | 3.62 | 0.059 |  | 1695.27 | 81.20 | 0 |
|  | intrinsic | BCI*Sex | 6 | 262.67 | 8.45 | 0.005 |  | 1697.41 | 83.35 | 0 |
|  | intrinsic + extrinsic (time) | Depart time*Chick age | 6 | 263.46 | 9.29 | 0.003 |  | 1614.06 | 0.00 | 1 |
|  | intercept | 0-model | 3 | 254.94 | 0.78 | 0.243 |  | 1693.23 | 79.17 | 0 |
| Rest | extrinsic (time) | Depart time | 4 | 303.29 | 18.04 | 0 |  | 2447.80 | 1.69 | 0.217 |
|  | extrinsic - time | Proportion ebb | 4 | 298.92 | 13.68 | 0.001 |  | 2505.82 | 59.71 | 0 |
|  | extrinsic (time) + extrinsic (weather) | Depart time*Wind speed | 6 | 297.32 | 12.07 | 0 |  | 2446.11 | 0.00 | 0.506 |
|  | extrinsic (weather) | Wind direction | 5 | 303.35 | 18.11 | 0 |  | 2475.76 | 29.65 | 0 |
|  | extrinsic - weather | Wind speed | 4 | 290.66 | 5.42 | 0.062 |  | 2497.90 | 51.79 | 0 |
|  | extrinsic - weather | Wind speed*Wind direction | 8 | 299.36 | 14.11 | 0 |  | 2449.22 | 3.11 | 0.107 |
|  | intrinsic + extrinsic (weather) | Wind speed*Chick age | 6 | 295.81 | 10.56 | 0.001 |  | 2501.10 | 54.99 | 0 |
|  | intrinsic + extrinsic (weather) | BCI*Wind speed | 6 | 285.25 | 0.00 | 0.002 |  | 2499.00 | 52.89 | 0 |
|  | intrinsic | Sex | 4 | 300.62 | 15.37 | 0 |  | 2507.61 | 61.50 | 0 |
|  | intrinsic | Chick age | 4 | 301.41 | 16.16 | 0 |  | 2508.21 | 62.10 | 0 |
|  | intrinsic | BCI | 4 | 296.69 | 11.44 | 0.003 |  | 2508.23 | 62.12 | 0 |
|  | intrinsic | BCI*Sex | 6 | 301.00 | 15.76 | 0.925 |  | 2509.21 | 63.10 | 0 |
|  | intrinsic + extrinsic (time) | Depart time*Chick age | 6 | 307.20 | 21.96 | 0.005 |  | 2448.28 | 2.17 | 0.171 |
|  | intercept | 0-model | 3 | 300.44 | 15.20 | 0 |  | 2507.00 | 60.89 | 0 |

**Appendix S4: Summary statistics of trip metrics**

**Table S4.1.** Summary statistics of the different trip parameters and distribution of habitats selected divided in colonies and years. Data are means (±SE. with n in parentheses)

| **Locality** | **Year** | **Variable** | **Coastal** | **Oceanic** |
| --- | --- | --- | --- | --- |
| Anda | 2011 | Duration (h) | 6.0 ± 0.43 (41) | 8.9 ± 1.23 (10) |
|  | 2012 | Duration (h) | 4.1 ± 0.99 (9) | 10.5 ± 1.23 (18) |
|  | 2013 | Duration (h) | 7.1 ± 0.41 (208) | 7.1 ± 0.41 (67) |
|  | 2014 | Duration (h) | 5.6 ± 0.32 (190) | 6.6 ± 0.29 (116) |
|  |  |  |  |  |
| Anda | 2011 | Path length (km) | 115.4 ± 9.06 (41) | 252.9 ± 34.09 (10) |
|  | 2012 | Path length (km) | 66.5 ± 20.17 (9) | 263.9 ± 27.65 (18) |
|  | 2013 | Path length (km) | 105.4 ± 3.97 (208) | 194.9 ± 9.77 (67) |
|  | 2014 | Path length (km) | 92.9 ± 5.00 (190) | 187.3 ± 6.61 (116) |
|  |  |  |  |  |
| Anda | 2011 | Maximum distance (km) | 34.5 ± 2.54 (56) | 76.4 ± 9.94 (13) |
|  | 2012 | Maximum distance (km) | 26.4 ± 8.60 (10) | 84.1 ± 9.14 (23) |
|  | 2013 | Maximum distance (km) | 38.7 ± 1.20 (221) | 61.8 ± 2.31 (68) |
|  | 2014 | Maximum distance (km) | 35.6 ± 1.78 (200) | 59.1 ± 1.75 (120) |
|  |  |  |  |  |
| Anda | 2011 | Number of trips (%) | 81 (56) | 19 (13) |
|  | 2012 | Number of trips (%) | 30 (10) | 70 (23) |
|  | 2013 | Number of trips (%) | 76 (221) | 24 (68) |
|  | 2014 | Number of trips (%) | 63 (200) | 27 (120) |
|  |  |  |  |  |
| Sør-Gjæslingan | 2011 | Duration (h) | 4.0 ± 2.07 (4) | - |
|  | 2012 | Duration (h) | 5.7 ± 0.72 (32) | 32.1 ± 4.10 (5) |
|  | 2013 | Duration (h) | 4.7 ± 0.34 (62) | 31.4 ± 3.09 (20) |
|  | 2014 | Duration (h) | 7.2 ± 0.61 (48) | - |
|  |  |  |  |  |
| Sør-Gjæslingan | 2011 | Path length (km) | 73.5 ± 36.4 (4) | - |
|  | 2012 | Path length (km) | 124.1 ± 19.75 (32) | 825.6 ± 40.02 (5) |
|  | 2013 | Path length (km) | 98.6 ± 9.68 (62) | 870.9 ± 52.04 (20) |
|  | 2014 | Path length (km) | 94.8 ± 9.24 (48) | - |
|  |  |  |  |  |
| Sør-Gjæslingan | 2011 | Maximum distance (km) | 21.3 ± 6.46 (9) | 290.8 ± 18.09 (5) |
|  | 2012 | Maximum distance (km) | 42.4 ± 8.20 (33) | 329.2 ± 8.28 (6) |
|  | 2013 | Maximum distance (km) | 27.6 ± 3.48 (66) | 299.5 ± 7.35 (21) |
|  | 2014 | Maximum distance (km) | 19.3 ± 1.39 (51) | - |
|  |  |  |  |  |
| Sør-Gjæslingan | 2011 | Number of trips (%) | 64 (9) | 36 (5) |
|  | 2012 | Number of trips (%) | 85 (33) | 15 (6) |
|  | 2013 | Number of trips (%) | 76 (66) | 24 (21) |
|  | 2014 | Number of trips (%) | 100 (51) | 0 |
